# The ageing microenvironment in sarcopenia: early alterations and targeted therapeutic strategies

**DOI:** 10.3389/fimmu.2026.1929891

**Published:** 2026-09-04

**Authors:** Liangping Zhang, Guangyang Li, Long Long, Lei Pan, Yun Bai, Ligang Ni, Linqiu Han, Rongqi Cao

**Affiliations:** 1 Hangzhou Linping District Hospital of Integrated Traditional Chinese and Western Medicine, Hangzhou, Zhejiang, China; 2 Sir Run Run Shaw Hospital, Zhejiang University School of Medicine, Hangzhou, Zhejiang, China

**Keywords:** ageing microenvironment, cellular senescence, early alterations, inflammaging, muscle regeneration, sarcopenia

## Abstract

Sarcopenia is a progressive skeletal muscle disorder associated with ageing and characterized by declines in muscle strength, mass and function. Increasing evidence suggests that its development is not driven solely by myofiber atrophy or impaired protein metabolism, but also by early remodeling of the ageing skeletal muscle microenvironment. This Review summarizes key early alterations in sarcopenia, including dysregulated immune homeostasis, cellular senescence and the senescence-associated secretory phenotype, depletion and dysfunction of muscle satellite cells, disruption of the regenerative niche, mitochondrial dysfunction, impaired proteostasis and neuromuscular junction degeneration. In aged skeletal muscle, chronic low-grade inflammation, oxidative stress, extracellular matrix stiffening and reduced regenerative capacity reinforce one another, establishing a self-amplifying cycle of inflammation, senescence and regeneration failure. This cycle may first impair muscle quality and contractile performance, resulting in early muscle weakness, and subsequently promote myofiber atrophy, fibrosis, fatty infiltration and overt functional decline. Accordingly, therapeutic strategies for sarcopenia should move beyond interventions aimed only at end-stage muscle loss and instead target the ageing microenvironment at earlier, potentially reversible stages. Potential approaches include exercise and nutritional interventions, modulation of inflammation, senescent cell clearance or suppression of the senescence-associated secretory phenotype, improvement of mitochondrial function and restoration of the muscle satellite cell niche. Biomarker- and omics-based population stratification may further support early detection, precision intervention and individualized management of sarcopenia.

## Highlights

- Ageing muscle microenvironment drives early sarcopenia progression.
- Inflammation, senescence and regeneration failure form a vicious cycle.
- MuSC depletion and niche disruption reduce regenerative capacity.
- Mitochondrial dysfunction and NMJ degeneration worsen muscle decline.
- Early microenvironment targeting may enable precision intervention.

## 1 Introduction

Sarcopenia is a progressive systemic skeletal muscle disorder primarily characterized by reduced muscle strength and decreased muscle mass or quality (1). With accelerating population ageing, the prevalence of sarcopenia is increasing, and the condition substantially reduces quality of life and healthspan in older adults. Worldwide, sarcopenia is estimated to affect more than 10% of individuals aged over 60 years, with an even higher prevalence among those aged over 80 years (2, 3). Sarcopenia is closely associated with reduced quality of life and increased risks of falls, fractures and mortality, making its early identification and comprehensive management an urgent priority in geriatric medicine and public health (4). Although exercise and nutritional interventions can improve muscle strength, physical function and nutritional status to some extent, they remain insufficient to fully reverse disease progression, highlighting the limitations of therapeutic strategies that target only end-stage muscle loss (5). Therefore, elucidating early remodeling mechanisms in the ageing muscle microenvironment and developing targeted interventions to restore impaired muscle regeneration are important for improving early detection and treatment of sarcopenia (6).

The pathophysiology of sarcopenia is highly complex and involves alterations at multiple cellular and molecular levels. Traditional studies have largely attributed sarcopenia to myofiber atrophy and imbalanced protein metabolism. However, growing evidence indicates that systemic disruption of the muscle microenvironment is not merely a secondary phenomenon, but an important mechanism that participates in, and may drive, the onset and progression of sarcopenia (7). During ageing, skeletal muscle undergoes diverse changes, including alterations in the transcriptomic landscape, dysregulation of signaling pathways, changes in the composition of myofibers and the extracellular matrix, disturbances in systemic metabolism and inflammatory responses, mitochondrial dysfunction and reduced neural input (8). Collectively, these changes create an ageing microenvironment that is unfavorable for the maintenance of muscle homeostasis. Among these processes, mitochondrial dysfunction and oxidative stress not only promote myofiber atrophy, but also exacerbate deterioration of the ageing muscle microenvironment by inducing inflammatory responses, cellular senescence and impaired proteostasis (9). In addition, chronic low-grade inflammation within the ageing microenvironment can promote the release of pro-inflammatory cytokines through innate immune signaling pathways, such as Toll-like receptors and the NLRP3 inflammasome, thereby further aggravating tissue damage and muscle atrophy (10). These findings suggest that sarcopenia cannot be explained solely by abnormalities in a single cell type or molecular pathway. Rather, it is more likely to result from an imbalanced microenvironmental network formed by interactions among myofibers, immune cells, stromal cells, neurovascular components, and soluble factors.

**Figure 1:**
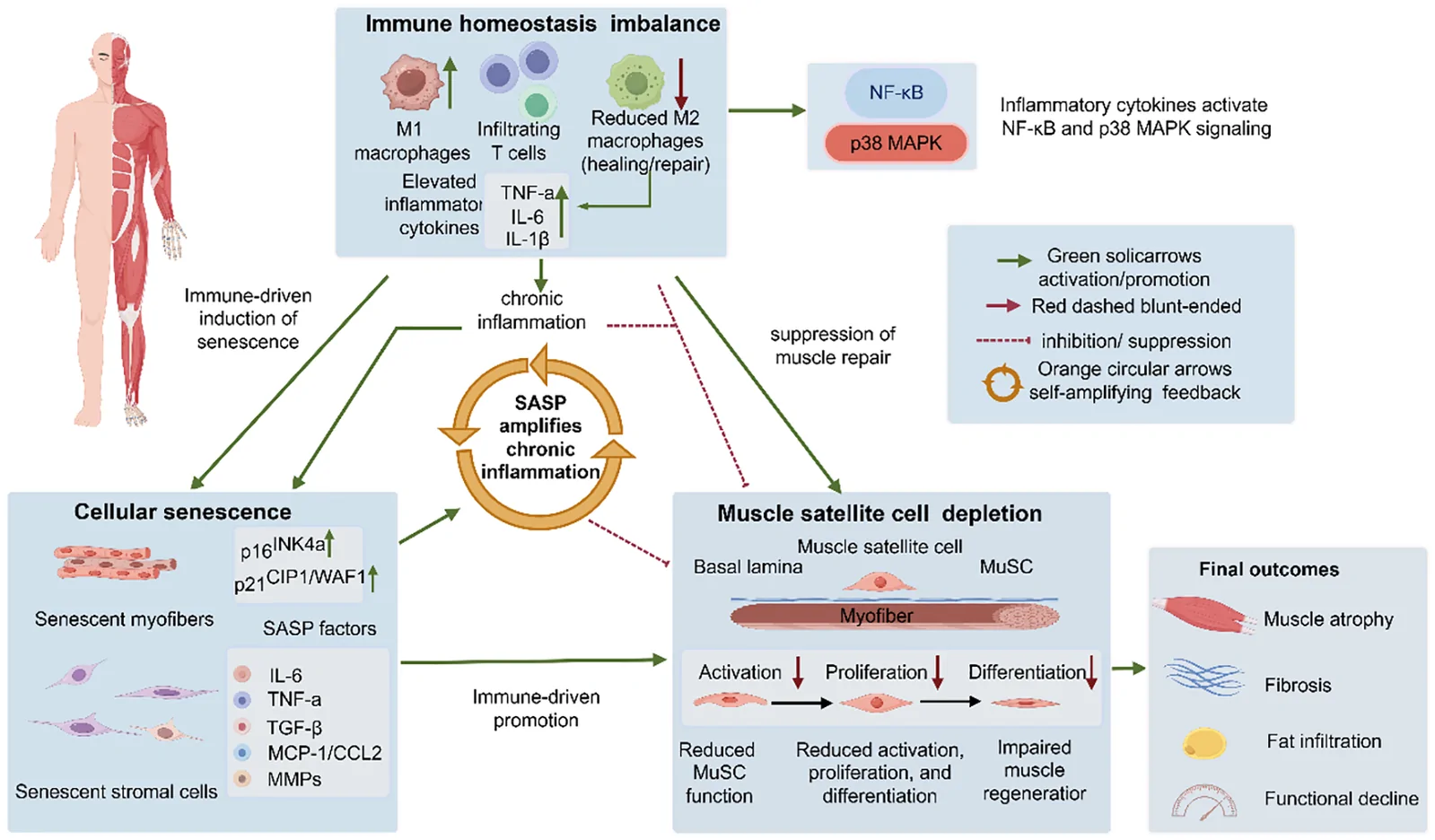
Interactions among immune dysregulation, cellular senescence and regenerative failure in the ageing skeletal muscle microenvironment. Ageing-related immune imbalance and chronic inflammation promote cellular senescence and SASP secretion, which further amplify inflammation and impair MuSC activation, proliferation and differentiation. This self-reinforcing cycle contributes to muscle atrophy, fibrosis, fatty infiltration and functional decline. Green arrows indicate activation, red dashed lines indicate inhibition, and circular arrows indicate feedback loops. By Figdraw (www.figdraw.com).

Early alterations in the ageing microenvironment may initially manifest as impaired muscle quality, reduced force-generating capacity and muscle weakness, before overt muscle mass loss becomes detectable (11). These functional changes can precede the later development of myofiber atrophy, fatty infiltration and fibrosis. In contrast to end-stage features such as myofiber atrophy, fatty infiltration and fibrosis, these early alterations emphasize antecedent processes, including immune inflammation, cellular senescence and disruption of the regenerative niche (12). Dysregulated immune homeostasis is mainly characterized by the establishment of chronic low-grade inflammation. Pro-inflammatory signaling can enhance muscle protein breakdown, suppress protein synthesis and weaken regenerative capacity (13). Through the SASP, senescent cells release pro-inflammatory factors, chemokines and mediators associated with matrix remodeling, thereby disrupting local microenvironmental homeostasis and further impairing the function of muscle satellite cells (MuSCs) (14, 15). Senescent cells can also establish a “geriatric-like inflammatory niche” during skeletal muscle regeneration, suppressing muscle stem cell proliferation and tissue repair. Meanwhile, MuSCs, the principal stem cell population responsible for skeletal muscle regeneration, undergo age-related declines in cell number, activation capacity and differentiation potential, leading to impaired repair after muscle injury (16). SASP factors released by senescent cells can amplify local inflammation, whereas chronic inflammation further induces senescence in neighboring cells and inhibits MuSC activation and proliferation. Together, these processes establish a positive feedback loop linking inflammation, senescence and regeneration failure (**Figure 1**) (17). If sustained, this process may drive the transition from early, potentially reversible alterations to muscle atrophy, fibrosis and functional decline. Accordingly, this Review focuses on the early pathological remodeling of the ageing microenvironment in sarcopenia, with particular emphasis on the roles of dysregulated immune homeostasis, cellular senescence, MuSC dysfunction and niche disruption in disease onset and progression. It further integrates the effects of metabolic abnormalities, impaired proteostasis and neurodegeneration on muscle homeostasis and regenerative capacity, with the aim of systematically delineating the regulatory roles of these pathological networks in the early progression of sarcopenia and exploring their potential as biomarkers for early detection and windows for targeted intervention.

## 2 Dysregulated immune homeostasis in the ageing microenvironment of sarcopenia

### 2.1 Aberrant innate immune responses

In aged skeletal muscle, aberrant innate immune responses represent an important early component of sarcopenic microenvironmental remodeling, although they should not necessarily be regarded as the primary or universally earliest pathological event. This abnormality is mainly characterized by impaired temporal switching of macrophage states and insufficient resolution of inflammation, which may drive the transition of muscle homeostasis from a compensatory state to a dysregulated state before overt muscle mass loss occurs (18). In healthy young muscle, macrophages dynamically shift between pro-inflammatory clearance-associated states and anti-inflammatory repair-associated states in response to injury and repair cues, thereby coordinating the removal of necrotic tissue, activation of muscle satellite cells and tissue regeneration (19). During ageing, this dynamic switching becomes metabolically and functionally constrained; in aged skeletal muscle, pro-inflammatory macrophages show impaired glycolytic reprogramming and phagocytic function during early recovery, which may delay inflammatory resolution and attenuate muscle regrowth (20). Ageing reduces macrophage responsiveness to interferon-γ, thereby weakening macrophage-supported satellite-cell proliferation and impairing skeletal muscle regeneration (21). These findings indicate that the early defect in aged muscle is not attributable to a single inflammatory mediator, but rather to disruption of the pro-resolving macrophage programs required to restore tissue homeostasis (22).

Macrophage dysfunction can sustain chronic low-grade inflammation in aged muscle, a process commonly referred to as “inflammaging”. During ageing, systemic and tissue-level chronic low-grade inflammation is characterized by modest but persistent increases in inflammatory mediators such as IL-6, TNF-α and IL-1β, creating an inflammaging milieu that predisposes the skeletal muscle microenvironment to inflammatory remodeling and functional decline (23, 24). In addition to inflammatory cytokines themselves, mitochondrial damage, oxidative stress, and defective clearance of cellular debris in aged muscle can result in an increased release of damage-associated molecular patterns (DAMPs) like ATP, mitochondrial DNA, HMGB1, and oxidatively modified proteins (25). The temporal relationship between tissue damage and immune dysregulation in sarcopenia is likely bidirectional rather than strictly linear. Age-related mitochondrial dysfunction and subclinical myofiber stress may emerge early, with mitochondrial deterioration preceding overt sarcopenic phenotypes in experimental models; damaged mitochondria and myofibers can also release DAMPs, including ATP, mitochondrial DNA and HMGB1, which are sensed by innate immune pathways involving TLRs and the NLRP3 inflammasome (26). Conversely, age-related defects in macrophage efferocytosis and inflammatory resolution may impair the clearance of damaged or apoptotic material and prolong damage-driven inflammatory activation (27). Persistent inflammatory signaling can subsequently impair mitochondrial function, increase oxidative stress and exacerbate myofiber injury, thereby reinforcing the inflammatory–metabolic disturbance (28). Thus, current evidence does not establish dysregulated immune homeostasis as a universally earliest pathological alteration; rather, tissue-intrinsic damage and immune dysregulation should be viewed as interacting, context-dependent early processes that can reciprocally reinforce the progression of sarcopenia (8). These endogenous danger signals can be sensed by innate immune recognition systems, including Toll-like receptors (TLRs) and the NLRP3 inflammasome, thereby promoting caspase-1 activation and the maturation and release of IL-1β and IL-18, which further sustain chronic inflammatory responses (29). Persistently elevated TNF-α, IL-6 and IL-1β can further activate inflammatory and catabolic pathways, including NF-κB, JAK/STAT and FoxO signaling, upregulate muscle atrophy-related factors such as MuRF1 and Atrogin-1 and enhance ubiquitin–proteasome system-mediated degradation of myofibrillar proteins. Skeletal muscle should also be considered an important amino acid reservoir during systemic inflammatory stress. Under acute conditions, inflammation-induced muscle protein mobilization represents an evolutionarily conserved adaptive response that provides amino acids for hepatic acute-phase protein synthesis, immune cell activation, tissue repair and gluconeogenesis (30). However, when inflammatory signaling persists, this normally beneficial metabolic adaptation may become maladaptive. Chronic activation of catabolic pathways, including NF-κB, JAK/STAT and FoxO signaling, continuously promotes muscle protein breakdown and gradually depletes skeletal muscle protein reserves. Thus, chronic inflammation may transform a transient protective response into a pathological driver of muscle wasting. In this way, early inflammatory signals are translated into catabolic responses associated with myofiber atrophy (31). In addition, inflammaging may link immune abnormalities to muscle metabolic dysfunction by affecting TRAF3-associated energy metabolism (32).

A persistent chronic inflammatory milieu not only promotes catabolic processes associated with myofiber atrophy, but also suppresses muscle regenerative capacity. Muscle satellite cells are the key stem cell population responsible for repair and regeneration after muscle injury, and their function depends on an inflammatory response that is transient, appropriately calibrated and resolved in a timely manner (33). In ageing, long-lasting pro-inflammatory mediators and abnormal macrophage responses interfere with satellite cell activation, proliferation and differentiation, shifting post-injury regeneration from effective repair to inefficient repair or even regenerative failure (34). At the same time, chronic inflammatory cues can shift fibro/adipogenic progenitors from transient pro-regenerative supporters toward persistent fibrogenic and adipogenic phenotypes, thereby promoting extracellular matrix deposition, intramuscular fat infiltration and disruption of the regenerative niche (35–37). This fibrotic replacement not only reduces muscle elasticity and contractile function but also further disrupts the satellite cell-mediated regenerative niche, forming a vicious cycle of inflammation, impaired regeneration and fibrosis. Thus, insufficient pro-resolving macrophage function, activation of the DAMP–TLR/NLRP3 inflammasome axis and persistent chronic low-grade inflammation may precede or accompany early declines in regenerative efficiency, progressively converting a potentially reversible immune microenvironmental imbalance into muscle protein catabolism, fibrotic remodeling and functional decline (38).

### 2.2 Aberrant adaptive immune responses

In the ageing muscle microenvironment, aberrant adaptive immune responses should not be regarded solely as a consequence of late-stage inflammation in sarcopenia; they may also participate in early immune homeostatic remodeling. T cell ageing and functional imbalance are key features of this process. With advancing age, thymic involution reduces the output of naive T cells, whereas the proportions of memory T cells, effector T cells and senescence-like T cells increase. This phenotypic shift compromises the ability of the immune system to recognize and eliminate new antigens, reflecting a decline in immune surveillance (39). Transcriptome-based immune deconvolution and histological validation of human skeletal muscle support an age- and sarcopenia-associated immune-remodeling pattern, with macrophage-dominant inflammation and altered communication among innate and adaptive immune cells in the sarcopenic muscle microenvironment (40). Therefore, the local accumulation of T cells, B cells and natural killer cells can be viewed as part of early microenvironmental network imbalance in sarcopenia, rather than merely as a secondary inflammatory phenomenon following end-stage myofiber atrophy (41). Accumulated effector T cells can produce pro-inflammatory mediators, such as IFN-γ and TNF-α, sustain a local pro-inflammatory state, and further amplify inflammaging and muscle catabolic signaling through interactions with macrophages, stromal cells and myofibers (42). Thus, T cell ageing is not simply a manifestation of systemic immunosenescence; through local infiltration, cytokine release and abnormal intercellular communication, it can drive persistent inflammation within the ageing skeletal muscle microenvironment.

Altered balance between regulatory T cells (Treg cells) and pro-inflammatory T cell responses represents another important feature of adaptive immune dysregulation. During normal muscle repair after injury, Treg cells not only suppress excessive inflammatory responses, but also promote tissue repair through amphiregulin, IL-10 and interactions with macrophages and satellite cells (43). In ageing, the recruitment, stability or reparative functions of local muscle Treg cells may be impaired, making it difficult for inflammation to transition in a timely manner toward resolution and regeneration (44). Recent studies indicate that Treg cells require IL-6 receptor α signaling to maintain skeletal muscle function and regenerative capacity; T cell-specific deletion of IL-6R impairs muscle Treg cell function and affects regeneration-associated cell populations, including satellite cells and fibro/adipogenic progenitors (45). At the same time, enhanced Th1, CD8^+^ effector T cell and certain IL-17-associated responses can exacerbate local inflammation through mediators such as IFN-γ, TNF-α and IL-17 (46). Notably, exercise-induced muscle Treg cells can limit IFN-γ-mediated mitochondrial damage and contribute to the protective effects of exercise on skeletal muscle metabolism and function. This finding suggests that Treg cells are not only immunosuppressive cells, but also important regulators of muscle metabolic homeostasis and the regenerative microenvironment (47). It further indicates that Treg cells may serve as early immune nodes for assessing whether the ageing muscle microenvironment retains interventional plasticity. Therefore, the key feature of adaptive immune dysregulation is not simply a change in the abundance of a single T cell subset, but rather an imbalance between pro-inflammatory effector T cell responses and the pro-resolving, pro-repair functions of Treg cells. This imbalance may delay the transition of inflammation into the regenerative phase and affect regeneration-associated cell populations, including satellite cells and fibro/adipogenic progenitors, at an early stage.

The roles of B cells and antibody responses in sarcopenia are receiving increasing attention, although the underlying mechanisms remain to be elucidated. During ageing, B cell generation and antibody repertoire diversity decline, whereas the propensity for autoreactive B cells and autoantibody production increases. These changes may provide a basis for abnormal humoral immunity in aged muscle (48). Recent studies have detected immunoglobulin G (IgG) infiltration in the skeletal muscle of some older adults, with IgG1 and IgG4 subclasses being particularly prominent. Muscle IgG1 levels are associated with poorer physical performance and reduced muscle strength (49). Further mechanistic studies suggest that these IgG antibodies may recognize cardiac troponin T (cTnT) and colocalize with markers of complement activation and apoptosis/necroptosis (50). Detection of anti-cTnT autoantibodies in aged mice and in human blood also suggests that cTnT-associated autoimmune responses may serve as potential biomarkers of ageing-related muscle injury (51). Overall, T cell infiltration, insufficient pro-repair Treg cell function and abnormal B cell/antibody responses may jointly prolong local inflammatory responses, impair the transition toward regeneration, and promote the progression of early, plastic immune imbalance toward persistent inflammation.

### 2.3 Immune-related signaling pathways

In early microenvironmental remodeling in sarcopenia, dysregulation of immune-related signaling pathways is a key link connecting danger signal recognition, persistence of chronic inflammation and initiation of catabolism. Among these pathways, NF-κB signaling is a central node linking inflammatory responses to muscle atrophy and can be persistently activated in aged muscle by TNF-α, IL-1β, reactive oxygen species (ROS) and DAMPs (52). Sustained NF-κB activation not only promotes the transcription of pro-inflammatory mediators such as TNF-α and IL-1β, but also upregulates muscle atrophy-related genes, including MAFbx and MuRF1, thereby enhancing ubiquitin–proteasome system-mediated degradation of muscle proteins (53). In addition, inflammation and oxidative stress form a self-reinforcing inflammatory–oxidative axis rather than acting as independent pathological processes. Chronic exposure to pro-inflammatory mediators, including TNF-α, IL-1β and IL-6, can impair mitochondrial homeostasis and promote mitochondrial ROS production and exacerbate cellular oxidative stress, whereas excessive ROS can reciprocally activate NF-κB- and NLRP3-dependent inflammatory signaling, thereby sustaining chronic low-grade inflammation (54, 55). At the myofiber level, this inflammatory–oxidative axis may contribute to functional impairment before overt muscle loss becomes detectable. Increased oxidative stress disrupts Ca²^+^ handling and excitation–contraction coupling and promotes oxidative modifications of sarcomeric and contractile proteins, thereby reducing myofilament responsiveness and intrinsic force-generating capacity (56, 57). With persistent inflammatory and oxidative stress, NF-κB, JAK/STAT and FoxO signaling converge on catabolic pathways, increasing MuRF1/Atrogin-1 expression and enhancing ubiquitin–proteasome- and autophagy–lysosome-mediated protein degradation (58). Thus, inflammaging may drive a temporal progression from mitochondrial oxidative stress and early contractile dysfunction to accelerated protein turnover, myofiber atrophy and subsequent muscle wasting.

The JAK/STAT signaling pathway, particularly the IL-6/JAK/STAT3 axis, is an important route through which inflammatory mediators influence muscle metabolism and regenerative capacity at early stages. In denervated muscle, FAPs can display persistent STAT3 activation and secrete high levels of IL-6, thereby promoting myofiber atrophy and fibrosis (59). In models of ageing- or disuse-associated muscle atrophy, Fyn is linked to the IL-6/STAT3–autophagy axis; Fyn deficiency reduces IL-6 levels and STAT3 phosphorylation while maintaining autophagic activity, suggesting that aberrant IL-6/STAT3 activation may contribute to early sarcopenia progression by impairing protein quality control (60). Thus, crosstalk between JAK/STAT3 and NF-κB can amplify inflammatory responses before overt muscle mass loss and, by affecting autophagic homeostasis, protein quality control and satellite cell differentiation, drive aged muscle toward an early pro-inflammatory, pro-catabolic and low-regenerative state.

The NLRP3 inflammasome is an important component of innate immune recognition systems. It can be activated by danger signals such as ATP, ROS and mitochondrial DNA, and promotes the maturation and release of IL-1β and IL-18 through caspase-1. IL-1β and IL-18 can exacerbate local inflammatory responses and may amplify inflammatory cascades through gasdermin D-mediated pyroptosis, further disrupting early muscle microenvironmental homeostasis (61). Studies indicate that the NLRP3 inflammasome is not restricted to immune cells; under inflammatory stimulation, it may also participate in morphological and metabolic abnormalities in skeletal muscle cells, including altered myotube morphology, increased mitochondrial ROS production and energy metabolic remodeling (55). Therefore, the NLRP3 inflammasome is not only an upstream regulatory node for inflammatory cytokine elevation, but may also directly contribute to metabolic abnormalities and morphological changes in skeletal muscle cells, serving as an important bridge that converts early immune-inflammatory signaling into myocellular injury.

Crosstalk between immune-inflammatory signaling and muscle protein degradation pathways constitutes an important molecular basis for muscle mass decline in sarcopenia. Age-associated increases in C4b can delay myogenic progenitor expansion and impair functional recovery, suggesting that complement dysregulation may contribute to microenvironmental remodeling at an early stage of declining regenerative efficiency (62). Pathways such as NF-κB, STAT3 and FoxO can jointly affect the ubiquitin–proteasome system, the autophagy–lysosome system and the IGF-1/PI3K/Akt/mTOR anabolic pathway, shifting aged muscle from a protein synthesis-dominant state toward a protein degradation-dominant state (58). Overall, NF-κB, JAK/STAT3, NLRP3, FoxO and complement-related signals represent important molecular nodes of early immune microenvironmental abnormalities. Before overt muscle mass loss occurs, these pathways can integrate danger signals, inflammatory mediators, oxidative stress, protein degradation and regenerative inhibition, progressively converting an initially targetable immune homeostatic imbalance into myofiber atrophy, fibrosis and functional decline (**Table 1**).

**Table 1:** Immune-cell dysregulation, associated signaling pathways and downstream pathological outcomes in early sarcopenia.

Immune cell/component	Immune alteration/key mediators	Signaling pathway	Downstream biological effect	Pathological outcome	Ref
Macrophages	Persistent pro-inflammatory state, impaired reparative/pro-resolving function; TNF-α, IL-6, IL-1β, DAMPs	TLR/NLRP3 → NF-κB; IL-6/JAK/STAT3; FoxO	Cytokine production; protein degradation; impaired MuSC regeneration	Chronic inflammation, atrophy and fibrosis	(10, 22, 29)
Effector T cells/Treg cells	Increased Th1/CD8^+^/IL-17-associated responses and reduced pro-repair Treg function; IFN-γ, TNF-α, IL-17	T-cell-associated inflammatory signaling/IL-6Rα-dependent Treg regulation	Persistent inflammation; impaired inflammatory resolution and MuSC support	Reduced regenerative capacity	(45, 63)
B cells/autoantibodies	Increased autoreactivity, IgG deposition and autoantibody production	Complement-associated signaling	Complement activation and muscle-cell injury	Muscle injury and impaired regeneration	(49, 62)
Shared inflammatory amplification	Persistent TNF-α, IL-1β, IL-6 and oxidative stress	NF-κB/JAK-STAT3/FoxO	Enhanced protein degradation; reduced anabolic signaling	Myofiber atrophy and functional decline	(31, 58)

## 3 Cellular senescence in the ageing skeletal muscle microenvironment

### 3.1 Senescence-like alterations across skeletal muscle-associated cell populations

Skeletal muscle ageing is a multicellular and multifactorial process. Senescence-like alterations across different cell types collectively constitute the cellular basis of early microenvironmental remodeling in sarcopenia. Myofibers are the principal effector units underlying skeletal muscle functional decline and may exhibit multiple ageing-associated features at relatively early stages. Studies have shown that genomic instability, accumulation of DNA damage, transcriptomic remodeling and impaired proteostasis can emerge early in aged skeletal muscle, and these alterations are closely associated with restricted maintenance of contractile proteins, reduced muscle mass and functional decline (64). Specifically, cell-cycle inhibitory molecules such as p16^INK4a^ and p21^CIP1^ can be upregulated in aged skeletal muscle, but their distribution is cell-type specific. This pattern suggests that some myofibers, fibro/adipogenic progenitors or other interstitial cells acquire senescence-like states, rather than indicating that all myofibers have entered irreversible growth arrest (65). Therefore, upregulation of p16^INK4a^ and p21^CIP1^ should be interpreted as an early signal of senescence-like alterations in specific cell populations, rather than evidence of terminal cellular senescence in all myofibers (66). In addition, aged myofibers are often accompanied by reduced mitochondrial oxidative capacity, increased ROS generation and impaired protein quality control. These changes can aggravate oxidative damage and proteostatic imbalance before overt myofiber atrophy and further promote myofiber wasting (67).

Senescence-like changes are also observed in regenerative and stromal cell populations within aged skeletal muscle. Muscle satellite cells (MuSCs) may shift from a stable quiescent state toward pre-activated or senescence-like states during ageing (68). Fibro/adipogenic progenitors (FAPs), which constitute an important stromal population in skeletal muscle, can likewise acquire ageing-associated phenotypic alterations (69, 70). In addition, endothelial and immune cells may develop senescence-like features that contribute to vascular and inflammatory abnormalities within the ageing muscle microenvironment (71). In summary, intrinsic myofiber damage can reduce contractile capacity and protein quality control, senescence-like changes in satellite cells can weaken regenerative reserves, and abnormalities in fibro/adipogenic progenitors, vascular cells and immune cells can further compromise local reparative support. Together, these changes drive early, potentially targetable cellular alterations toward myofiber atrophy, fibrosis and functional decline.

### 3.2 SASP-mediated remodeling of the ageing muscle microenvironment

The senescence-associated secretory phenotype (SASP) refers to a broad array of bioactive factors released by senescent cells and represents a key secretory mechanism through which early cellular senescence affects the skeletal muscle microenvironment in sarcopenia. The SASP can disrupt muscle microenvironmental homeostasis through multiple mechanisms and promote the emergence of a low-regenerative, pro-inflammatory and pro-fibrotic state before overt muscle mass loss occurs. Core SASP components include IL-6, TNF-α, IL-8, CCL2/MCP-1 and various matrix metalloproteinases, all of which are upregulated during ageing and can sustain local and systemic chronic low-grade inflammation (72). Evidence suggests that chronic low-grade inflammation mediated by inflammatory factors such as IL-6 and MCP-1 can remodel the skeletal muscle microenvironment before overt muscle loss, representing an important early mechanism in the onset and progression of sarcopenia (73). In addition, SASP factors can enhance inflammatory signaling, promote catabolism and weaken anabolic responses such as IGF-1/Akt/mTOR signaling, thereby shifting the balance between protein synthesis and degradation towards degradation (74). Thus, SASP factors released by senescent cells can sustain local inflammatory responses and affect protein metabolism, immune cell recruitment and satellite cell function before marked myofiber atrophy becomes evident.

SASP-mediated remodeling of the extracellular matrix (ECM) is another key mechanism linking early cellular senescence to subsequent muscle fibrosis and matrix stiffening. Senescent cells can alter the balance of ECM degradation, deposition and crosslinking by releasing MMPs, TGF-β, chemokines and pro-fibrotic factors (75). An aged-like inflammatory regenerative niche can suppress stem-cell proliferation and muscle regeneration through inflammation- and fibrosis-associated SASP programs (76). In addition, LOXL2 inhibitors can attenuate D-gal-induced skeletal muscle fibrosis and partially improve muscle mass and strength, suggesting that ECM crosslinking-related alterations may represent a potential intervention point through which the SASP links early matrix remodeling to subsequent functional decline (77). Single-cell and single-nucleus multi-omic atlases of ageing human skeletal muscle have revealed remodeling of communication networks among FAPs, immune cells, vascular cells and satellite cells, accompanied by enhanced inflammatory responses, matrix remodeling and molecular features associated with sarcopenia susceptibility (78). Thus, the SASP does not merely increase the release of inflammatory factors but also promotes broader inflammatory, matrix and intercellular communication abnormalities that create a microenvironment increasingly unfavorable for effective muscle regeneration.

**Figure 2:**
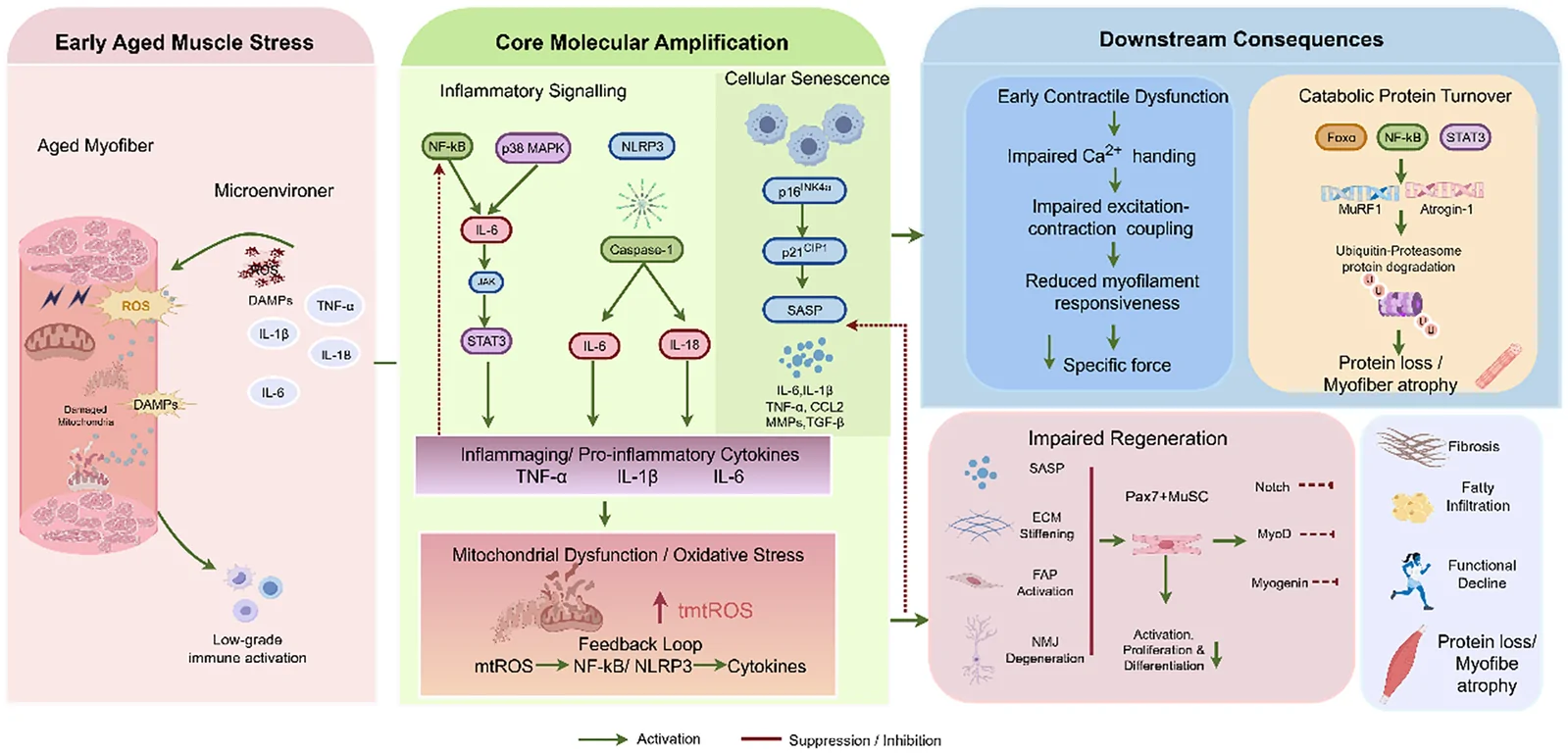
Early pathological remodeling of the ageing skeletal muscle microenvironment. Chronic inflammatory signaling promotes mitochondrial dysfunction and oxidative stress, which in turn reinforce inflammatory pathways and contribute to early contractile dysfunction through impaired Ca²^+^ handling, excitation–contraction coupling and oxidative modification of contractile proteins. Persistent inflammatory–oxidative signaling further activates NF-κB-, JAK/STAT- and FoxO-dependent catabolic pathways, enhancing ubiquitin–proteasome- and autophagy-mediated protein degradation and ultimately promoting myofiber atrophy. These processes interact with cellular senescence, impaired regeneration, FAP activation and NMJ degeneration to drive sarcopenia progression. By Figdraw (www.figdraw.com).

Paracrine propagation of senescence signals is an important feature of the SASP, enabling senescence-like states to spread within local tissues and amplify early pathological effects. ROS, cell-free DNA, chemokines, proteases and extracellular vesicles carrying specific proteins and nucleic acids released by senescent cells can act on neighboring myofibers, satellite cells, FAPs and immune cells, inducing secondary inflammatory responses, senescence-like alterations or regenerative inhibition (79). Reducing senescent-cell burden or suppressing the inflammatory secretome of senescent cells can improve skeletal muscle function and reduce senescence-associated markers in aged muscle, suggesting that cellular senescence is not merely a passive marker of ageing but a modifiable therapeutic target in early sarcopenia (80). Collectively, cellular senescence and SASP-mediated signaling contribute to an inflammatory and structurally altered ageing muscle microenvironment (**Figure 2**).

## 4 Muscle satellite cell dysfunction and regenerative niche disruption in sarcopenia

### 4.1 Decline in the effective muscle satellite cell reserve

Muscle satellite cells (MuSCs) are adult stem cells responsible for maintaining skeletal muscle balance and facilitating repair following injury. A reduction in their effective reserve and mobilization capacity represents an important cellular alteration underlying the early decline in regenerative capacity in sarcopenia (81, 82). During ageing, the effective MuSC pool may gradually decline, largely owing to senescence-like changes, increased apoptosis and impaired self-renewal capacity (83, 84). Cross-age single-cell and spatial transcriptomic studies have shown that aged MuSCs exhibit reduced self-renewal capacity and an increased prevalence of pre-senescent states, leading to progressive contraction of the MuSC pool required for regeneration (85). These intrinsic alterations, together with microenvironmental dysregulation, drive age-dependent MuSC dysfunction, weakening their regenerative competence and thereby aggravating early regenerative defects in sarcopenia (86).

Mitochondrial dysfunction and oxidative stress can further exacerbate cellular damage and reduce the effective satellite cell reserve (87). Abnormal exit from quiescence reduces the number of functionally mobilizable stem cells in the satellite cell pool during injury, thereby diminishing muscle regenerative potential. Even when the total number of MuSCs has not yet markedly declined, their mobilization, migration and local expansion after muscle injury may already be impaired, reducing the number of cells effectively participating in repair. This decline in mobilization and recruitment is closely linked to early remodeling of the ageing muscle microenvironment. Ageing-related changes in mechanical cues, nutrient supply and paracrine support can weaken MuSC migration, localization, activation and expansion (88, 89). Maintenance of quiescence is essential for the long-term stemness and regenerative reserve of satellite cells. Ageing can cause satellite cells to exit normal quiescence abnormally and prematurely enter senescence-like or pre-activated states, thereby compromising their timely response to muscle injury (90). Overall, senescence-like alterations, increased apoptosis, insufficient self-renewal, impaired mitochondrial quality control and defective mobilization together reduce the quiescent Pax7+ MuSC reserve and the effectively mobilizable cell pool, causing skeletal muscle regenerative capacity to decline before overt atrophy becomes apparent.

### 4.2 Intrinsic functional decline of muscle satellite cells

As the principal adult stem cells in skeletal muscle, MuSCs may undergo functional decline across a continuum of injury sensing, activation, proliferation, differentiation and self-renewal. This decline constitutes an important cellular basis for the early reduction in regenerative reserve in sarcopenia (91). MuSC function includes not only post-injury activation, proliferation and myogenic differentiation, but also self-renewal and return to the quiescent pool, which are required to maintain long-term regenerative capacity (92).

During ageing, MuSC functional decline first manifests as impaired injury responsiveness and activation. Under normal conditions, after muscle injury, MuSCs sense injury-associated signals in the microenvironment and rapidly transition from quiescence to activation. This process involves early stress responses, initial ERK1/2 proliferative signaling and Notch-regulated myogenic programs (93). In aged skeletal muscle, however, MuSCs exhibit reduced sensitivity to injury signals and decreased Notch pathway activity, impairing their transition from quiescence to activation (94). This activation defect can reduce the efficiency of regenerative initiation after injury before overt muscle mass loss occurs, leading to delayed repair responses and insufficient regenerative output in aged skeletal muscle (95). Importantly, the functional defects of aged MuSCs retain a degree of microenvironmental plasticity: exposure to a young niche can reverse approximately half of the age-related transcriptional alterations in aged MuSCs, suggesting that early functional decline is not entirely fixed (96).

Second, even when some aged MuSCs are successfully activated, their proliferative expansion may be impaired. Activated MuSCs must expand through cell division to generate sufficient myogenic progenitors for muscle repair (97). However, aged MuSCs may proliferate more slowly and exhibit prolonged cell-cycle progression. This phenomenon is closely associated with upregulation of cell-cycle inhibitory proteins such as p16INK4a and p21Cip1, accumulation of DNA damage and enhanced inflammatory SASP signaling. Studies have shown that aged satellite cells can exhibit G0/G1 cell-cycle arrest, increased DNA damage and elevated senescence-associated β-galactosidase activity (98). Declining activity of the hypothalamic–pituitary–gonadal axis can impair autophagosome clearance in satellite cells by suppressing transcription factor EB expression, thereby accelerating senescence-like changes (99). In parallel, abnormalities in mitochondrial dynamics and quality control can also weaken MuSC proliferation and regenerative output. Impaired DRP1-associated mitochondrial fission reduces satellite cell regenerative capacity, indicating that mitochondrial homeostasis is an important intrinsic mechanism linking ageing to MuSC functional decline (100).

In addition to impaired activation and proliferation, reduced myogenic differentiation of aged MuSCs should be understood as a disruption in early regenerative program switching. In this state, activated cells fail to efficiently upregulate myogenic transcriptional programs, such as MyoD and Myogenin, and fail to form mature myotubes (101). Myogenic differentiation is a critical step through which satellite cells ultimately form multinucleated myotubes and repair damaged myofibers; this process is tightly regulated by myogenic transcription factors, including MyoD and Myogenin (102). In aged MuSCs, the expression of these key transcription factors may decline, reducing the efficiency of differentiation into myotubes. Dysregulated FOXO3 signaling can affect Myod1 and Myog expression, reduce the efficiency of myoblast-to-myotube conversion, decrease myotube size and weaken responses to electrical stimulation, suggesting that impaired myogenic differentiation may serve as an early functional indicator of MuSC decline (103). Taken together, MuSCs already exhibit insufficient injury sensing, delayed activation, reduced effective expansion, impaired myogenic differentiation and limited self-renewal before overt muscle mass loss occurs, and these defects progressively develop into persistent regenerative insufficiency under the influence of the ageing microenvironment.

### 4.3 Extrinsic disruption of the muscle satellite cell regenerative niche

The MuSC niche is composed of myofibers, fibro/adipogenic progenitors (FAPs), immune cells, vascular cells, nerve-associated cells, the extracellular matrix (ECM) and soluble factors. Early disruption of niche homeostasis can first affect MuSC activation, migration, proliferation, differentiation and self-renewal, before later manifesting as myofiber atrophy, fibrosis and fatty infiltration (104).

Aberrant activation of FAPs is a key event in early MuSC niche alteration. In young muscle, FAPs proliferate transiently after injury, support MuSC differentiation and are subsequently cleared by apoptosis. However, in the contexts of ageing, chronic inflammation or denervation, FAPs can shift from a transient pro-regenerative state to a persistently activated state with pro-fibrotic and pro-adipogenic tendencies, thereby weakening the regenerative niche of MuSCs before overt structural damage occurs (105). Senescence-related abnormalities within FAPs may further compromise their niche-supporting function. For example, oxidative stress-mediated senescence of FAPs can disturb their pro-regenerative support for MuSCs and shift the post-injury niche toward impaired repair (88). In addition, Pim1 knockout reduces adipogenic differentiation of PDGFRα+ mesenchymal progenitors by inhibiting the C/EBPδ pathway, thereby alleviating intramuscular adipose tissue accumulation and sarcopenic phenotypes in aged mice (70). Studies have demonstrated important paracrine communication between FAPs and MuSCs; for example, the FGF7–FGFR2 axis promotes satellite cell proliferation. These findings suggest that FAPs are not intrinsically detrimental; rather, loss of temporal control and pathological differentiation of FAPs are central to abnormalities of the ageing niche (106). Such aberrant FAP behavior disrupts the precise regulation of muscle regeneration, initially suppressing MuSC activation and function and subsequently promoting fibrosis and fatty infiltration.

At the same time, ECM stiffening and fibrotic tendency constitute another early feature of abnormalities in the ageing muscle niche. During ageing, increased collagen deposition and matrix stiffness can alter the mechanical signals perceived by MuSCs (107). This abnormal mechanical microenvironment can suppress MuSC activation and differentiation before overt muscle mass loss, making these cells more prone to functional dysfunction (108). Proteomic studies have shown extensive ECM remodeling in MuSCs and their niche in aged muscle. Increased FAP-derived Smoc2 can disrupt signaling axes such as integrin β1/MAPK, thereby impairing MuSC function and muscle regenerative capacity (109). In addition, ECM stiffening can form a vicious cycle with the pro-fibrotic transformation of FAPs, jointly aggravating niche imbalance. Degeneration of the vascular microenvironment is another important source of early MuSC niche dysfunction. In aged muscle, reduced capillary density and impaired endothelial function can limit the supply of oxygen, nutrients and paracrine signals around MuSCs (110). Human skeletal muscle ageing atlases have also shown reductions in niche cells, including vascular cells and Schwann cells, accompanied by immune cell recruitment and changes in NMJ-associated myonuclear populations. The hypoperfused and hypoxic microenvironment resulting from vascular degeneration may be an important driver of MuSC functional decline (11). Therefore, hypoperfusion and weakened endothelial–myogenic interactions caused by vascular degeneration may represent important contributors to early impairment of the MuSC regenerative niche.

Early degeneration of the neuromuscular junction (NMJ) is an important aspect of MuSC niche dysfunction. During ageing, loss of motor neurons and disruption of NMJ structure can lead to myofiber denervation. This not only weakens muscle function, but may also affect MuSCs by altering the myofiber secretome. Denervation can remodel the myofiber secretome and increase factors such as osteopontin and TGF-β1, thereby prematurely altering MuSC lineage progression and regenerative function. These findings suggest that NMJ-related changes can influence the regenerative niche before overt muscle mass loss occurs (111). In summary, loss of temporal control in FAPs, ECM stiffening, vascular degeneration and early NMJ deterioration collectively constitute major sources of MuSC niche impairment in aged muscle. Through mutually reinforcing inflammatory responses, pro-fibrotic remodeling, hypoperfusion and denervation-related signaling, these processes drive the progression of sarcopenia from early regenerative dysfunction toward muscle atrophy, fatty infiltration and functional decline.

## 5 Interactions among key factors in the ageing microenvironment of sarcopenia

### 5.1 Inflammation and cellular senescence

During the pathogenesis of sarcopenia, dysregulated immune homeostasis and cellular senescence engage in bidirectional regulation and can remodel local skeletal muscle microenvironmental homeostasis before overt muscle mass loss and severe functional decline occur. The central feature of this stage is not end-stage myofiber atrophy, but the coexistence of chronic low-grade inflammation, persistent SASP secretion, insufficient clearance of senescent cells and suppression of the regenerative niche. Together, these alterations drive skeletal muscle into a low-regenerative and pro-catabolic state before marked structural damage becomes apparent (112). Chronic low-grade inflammation, or inflammaging, can promote senescence-like alterations in skeletal muscle-associated cells. Inflammatory mediators such as TNF-α and IL-6 can promote changes related to p16INK4a, p21CIP1 and the SASP through p38 MAPK, NF-κB and DNA damage responses (113). At the same time, inflammatory signaling can disrupt protein metabolic balance before structural muscle loss occurs by activating catabolic pathways and suppressing protein synthesis (114). Thus, inflammation not only contributes directly to myofiber dysfunction but also facilitates the establishment of a senescence-prone microenvironment.

Conversely, senescent cells can become an important source of persistent inflammation through SASP secretion. Senescence-like myogenic cells, FAPs and immune cells can secrete SASP factors, including IL-6, TNF-α, CCL2, CXCL-family chemokines and matrix metalloproteinases. These factors further activate NF-κB, p38 MAPK and JAK/STAT pathways, intensify inflammatory responses and promote immune cell infiltration (115). This senescent cell-driven inflammatory milieu can, in turn, induce senescence-like alterations in neighboring cells, thereby establishing a positive feedback loop in which inflammation promotes senescence and senescence further amplifies inflammation (116). Recent single-cell studies further show that senescent cells in injured muscle can form an inflammatory niche resembling that of aged muscle and suppress skeletal muscle regeneration at multiple stages, suggesting that the SASP functions as an important amplifier connecting senescence with inflammation and regenerative dysfunction (117).

Declining immune-mediated clearance of senescent cells further strengthens this feedback loop. With advancing age, immune cells themselves undergo ageing, a process referred to as immunosenescence. An aged immune system can promote cellular senescence and tissue damage in non-lymphoid organs, suggesting that impaired immune surveillance may prevent the timely removal of senescence-like cells that emerge in skeletal muscle (118). Their persistent accumulation sustains SASP secretion and maintains a chronic inflammatory microenvironment. Therefore, inflammation and senescence should be viewed as mutually reinforcing processes rather than separate abnormalities, and their interaction provides an important mechanistic link to the subsequent decline in regenerative capacity.

### 5.2 Cellular senescence and impaired regeneration

The inflammation–senescence loop is closely coupled to impaired muscle regeneration. Through the SASP, senescent cells can create inflammatory and matrix-remodeling signals that impair MuSC activation, expansion and differentiation, thereby shifting regeneration toward inefficient repair rather than complete myofiber restoration (119). Studies have shown that knockdown of p21 (Cdkn1a) in *in vitro*-induced senescence-like myogenic cells not only reduces the number of senescent cells, but also decreases IL-6 and TNF-α expression and restores myogenic differentiation capacity, suggesting that suppression of senescence-associated inflammatory signaling may help restore myogenic function (120). More importantly, single-cell transcriptomic and senescent cell-enrichment analyses show that senescent cells in injured muscle can establish a “geriatric-like inflammatory niche” characterized by inflammatory and pro-fibrotic features, suppressing stem cell proliferation and muscle regeneration during early repair. Reducing senescent cell burden or suppressing their inflammatory secretome promotes muscle regeneration in both young and aged mice, indicating that senescent cells and their SASP represent targetable components of early regenerative impairment.

Senescence-like alterations in MuSCs themselves can further amplify this process. MuSCs with senescence-like changes not only exhibit impaired self-renewal and myogenic capacity, but some may also secrete SASP factors that aggravate local inflammation and pro-fibrotic remodeling (121). For example, CCN2, also known as connective tissue growth factor, is increasingly secreted by aged MuSCs, whereas long-term aerobic exercise can suppress this process, thereby improving muscle regeneration and reducing fibrosis (122). In addition, mitochondrial dysfunction and metabolic abnormalities in aged MuSCs, such as reduced glutamine-driven reductive tricarboxylic acid cycle flux, can further weaken their self-renewal and regenerative capacity (123). These changes establish a reciprocal relationship between senescence and regenerative failure. Senescence and SASP signaling impair MuSC-mediated repair, whereas incomplete regeneration permits damaged myofibers, inflammatory signals and pro-fibrotic niche alterations to persist, thereby creating conditions that further promote cellular stress and senescence. This vicious cycle reduces the effective MuSC reserve, promotes chronic inflammation and pro-fibrotic remodeling, and ultimately drives the progression from early regenerative impairment to sarcopenia (124, 125). Therefore, early regenerative failure should not be regarded simply as a downstream consequence of senescence, but as an active component of the self-reinforcing pathological network. Cross-age single-cell and spatial transcriptomic studies further show that aged muscle injury sites can exhibit senescence-associated MuSC states and abnormal regenerative trajectories, suggesting that early regenerative impairment arises not only from reduced MuSC numbers but also from disrupted post-injury cell-state transitions. Moreover, glutamine-driven reductive TCA cycling and the lipid synthesis it mediates can support aged MuSC function, indicating that insufficient metabolic adaptability may further constrain MuSC self-renewal and regenerative output and link regenerative failure to metabolic injury.

### 5.3 Metabolic injury, proteostatic imbalance and neurodegeneration

Metabolic injury, proteostatic imbalance and neuromuscular junction degeneration further amplify the inflammation–senescence–regeneration network. Mitochondrial dysfunction in aged muscle is closely linked to chronic inflammation. Persistent exposure to inflammatory cytokines can aggravate mitochondrial damage and ROS generation, while mitochondrial ROS and damage-associated signals can further activate inflammatory pathways, establishing a bidirectional inflammation–mitochondrial dysfunction–oxidative stress loop. The resulting decline in energy metabolism can weaken myofiber homeostasis before overt muscle mass loss and create a metabolic basis for subsequent myofiber atrophy. At early stages, mitochondrial alterations should be understood primarily as reduced oxidative phosphorylation efficiency, increased ROS production, abnormal mitochondrial dynamics and impaired mitophagy, rather than being equated directly with established myofiber atrophy (126). Aged skeletal muscle can exhibit sex-related mitochondrial proteomic remodeling, suggesting that mitochondrial injury may be an important dimension of sarcopenic microenvironmental heterogeneity and early metabolic risk stratification (127). Insufficient energy supply can activate energy sensors such as AMPK, thereby inhibiting mTOR-mediated protein synthesis and promoting FOXO-dependent activation of the ubiquitin–proteasome system and autophagy–lysosome system, shifting proteostasis toward degradation at an early stage (128). Studies of chronic wasting conditions, such as heart failure with preserved ejection fraction and cancer cachexia, show that skeletal muscle can exhibit reduced mitochondrial respiratory function, impaired oxidative phosphorylation and imbalanced mitochondrial dynamics. These changes are closely associated with reduced exercise tolerance, muscle mass loss and myofiber atrophy (129, 130). Defective mitochondrial and proteostatic quality control can in turn generate additional cellular stress and further reinforce inflammation and senescence. TP53INP2/TRP53INP2 is reduced in aged skeletal muscle in humans and mice, and its muscle-specific upregulation can improve muscle atrophy in aged mice, enhance mitophagy and reduce ROS (131). In parallel, age-related decline of chaperone-mediated autophagy in skeletal muscle reduces proteostatic and mitochondrial homeostasis and leads to progressive myopathy, whereas genetic CMA enhancement in old mice partially ameliorates ageing-associated muscle phenotypes (132). Thus, metabolic injury and proteostatic imbalance are not isolated downstream consequences, but important amplifiers that sustain inflammatory and senescence-associated signaling while limiting regenerative capacity.

Neuromuscular junction (NMJ) degeneration represents another component of this interconnected network. Motor-unit and NMJ abnormalities in ageing and sarcopenia can be detected using electrophysiological approaches and are associated with reduced muscle strength and impaired motor function. Therefore, NMJ dysfunction should be considered an important component of early neuromuscular microenvironmental remodeling in sarcopenia (133). Motor neuron-specific deletion of Prmt1 can lead to age-related motor neuron degeneration, NMJ dysfunction and muscle loss, accompanied by mitochondrial dysfunction and increased cellular stress, suggesting a mechanistic link between NMJ degeneration and metabolic injury (134). Human sarcopenia data indicate that neuromuscular impairment is detectable across sarcopenia stages by motor-unit and NMJ assessments and tracks with poorer muscle performance, supporting NMJ dysfunction as an early, clinically relevant neuromuscular component rather than a late by-product of myofiber loss (135).

The vulnerability of type II fast-twitch fibers further illustrates the interaction between neuromuscular and metabolic injury. Compared with slow-twitch type I fibers, fast-twitch type II fibers have lower mitochondrial content and oxidative capacity and rely more heavily on glycolytic metabolism. They are therefore more sensitive to energy metabolic disturbances and oxidative stress. During ageing, type II fibers may exhibit earlier functional vulnerability and subsequently undergo preferential atrophy, a process associated with motor neuron loss, insufficient reinnervation and proteostatic imbalance (136). Because fast-twitch fibers have limited mitochondrial reserves, they are less tolerant of insufficient ATP production and ROS accumulation and are therefore more prone to activating protein degradation pathways. Thus, their early vulnerability and subsequent preferential atrophy reflect the synergistic effects of mitochondrial dysfunction, proteostatic stress and NMJ degeneration and may help explain the early decline in muscle strength and explosive power observed in sarcopenia (137).

Early functional impairment in ageing skeletal muscle should not be equated with loss of muscle mass. Accumulating evidence from ageing and chronic inflammatory conditions suggests that muscle strength and force-generating capacity may decline before overt reductions in muscle mass become detectable (138, 139). This dissociation between muscle quantity and muscle quality indicates that contractile dysfunction may represent an early manifestation of microenvironmental dysregulation (140). At the myofiber level, age-related disruption of Ca²^+^ homeostasis and excitation–contraction coupling provides an important mechanism for this early loss of force. Alterations in the T-tubule–sarcoplasmic reticulum Ca²^+^ release machinery, including impaired Cav1.1–RyR1 coupling, dysregulated RyR1-mediated Ca²^+^ release and defective sarcoplasmic reticulum Ca²^+^ reuptake, can reduce the amplitude and fidelity of intracellular Ca²^+^ signals required for myofilament activation (56, 141). Consequently, membrane excitation may be translated less efficiently into Ca²^+^ release and mechanical force even before substantial myofiber atrophy develops. Chronic inflammation and mitochondrial dysfunction may further aggravate this process by limiting ATP availability and increasing oxidative stress, thereby disrupting Ca²^+^ handling and contractile homeostasis. In parallel, excessive oxidative stress can induce redox post-translational modifications of sarcomeric and cytoskeletal proteins. In this context, chronic inflammatory signaling can act upstream of mitochondrial oxidative stress, thereby linking inflammaging directly to impaired Ca²^+^ handling, excitation–contraction coupling and redox modification of contractile proteins. Oxidative modification of proteins involved in force transmission and contraction can impair myofilament function, alter contractile efficiency and reduce intrinsic force-generating capacity independently of changes in muscle size (57). These alterations may therefore produce clinically relevant muscle weakness before substantial myofiber atrophy develops, positioning reduced contractile performance as a potentially earlier consequence of inflammaging than overt sarcopenic muscle wasting (142).

**Figure 3:**
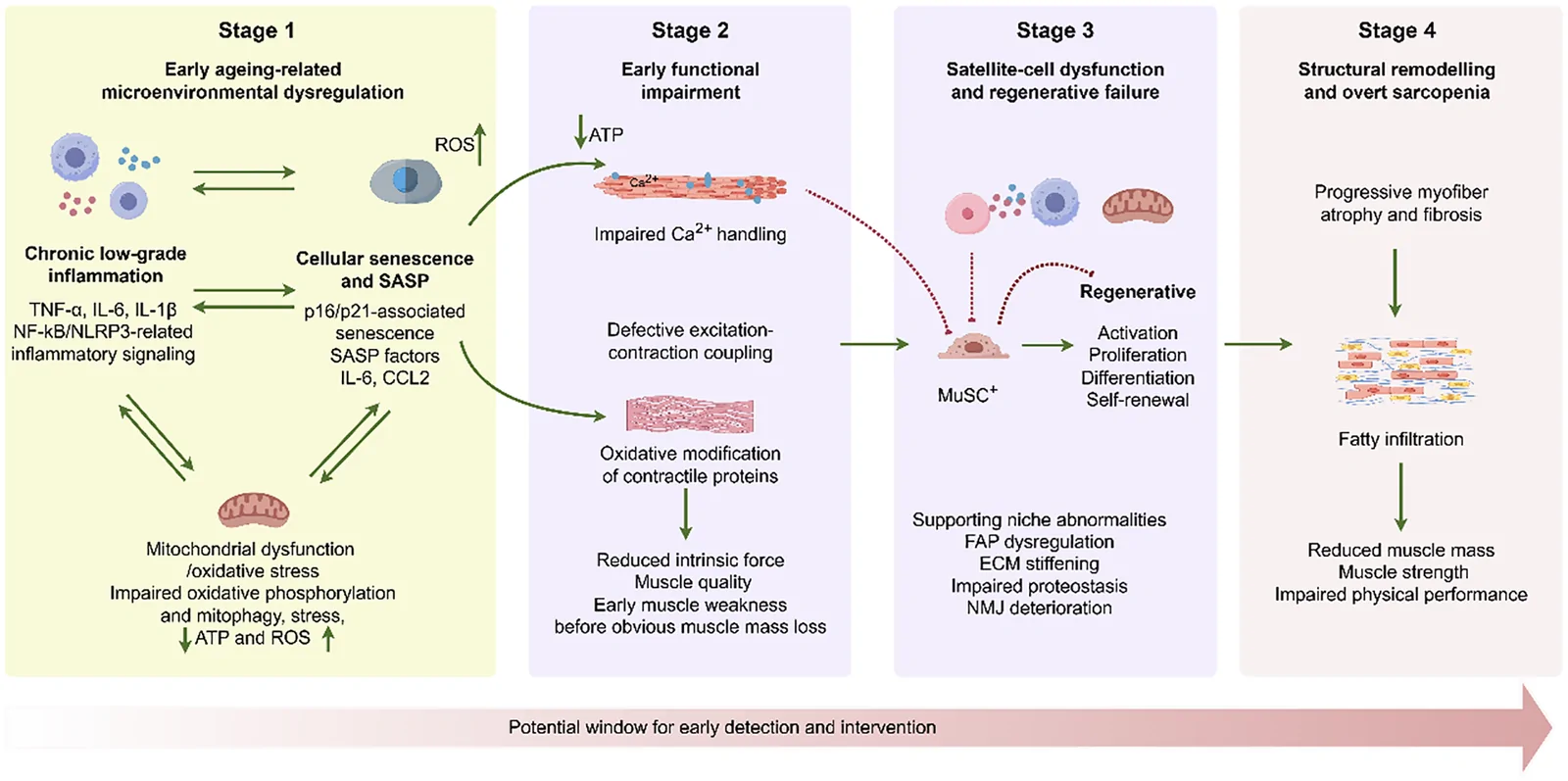
Temporal progression of sarcopenia. Ageing-related inflammation, cellular senescence/SASP and mitochondrial dysfunction interact to cause early muscle dysfunction, followed by satellite-cell impairment, regenerative failure, structural remodeling and overt sarcopenia. These processes are interconnected through self-amplifying feedback loops. By Figdraw (www.figdraw.com).

Overall, the progression of sarcopenia is not driven by a single factor, but by multilevel positive feedback loops formed through the interaction of inflammation, cellular senescence, regenerative failure and metabolic dysfunction within the ageing microenvironment. SASP factors can induce paracrine senescence, amplify inflammatory responses and promote tissue dysfunction (143). Inflammatory mediators, oxidative stress and extracellular matrix (ECM) stiffening impair satellite cell activation, proliferation and differentiation (144), while impaired mitochondrial quality control and proteostasis increase ROS accumulation and reduce energy availability. In turn, defective regeneration permits damaged myofibers and abnormal stromal remodeling to persist, further sustaining inflammatory and senescence-associated signals. As these feedback loops become chronic, excessive ECM deposition and fibrosis increase muscle stiffness and impair interactions between satellite cells and myofibers as well as nutrient diffusion (145). At the same time, increased differentiation of FAPs into adipocytes promotes intermuscular adipose tissue infiltration, and these adipocytes secrete pro-inflammatory adipokines that further exacerbate local inflammation (146). Therefore, breaking this vicious cycle requires early identification and simultaneous targeting of multiple interacting nodes, rather than focusing solely on a single end-stage phenotype (**Figure 3**).

## 6 Heterogeneity of the ageing microenvironment in sarcopenia

### 6.1 Population heterogeneity

Early alterations in the ageing microenvironment of sarcopenia do not follow a uniform pattern across all patients; rather, they are jointly shaped by age, sex, biological ageing status, nutritional and metabolic conditions, and physical activity level (147). Patients at different stages of ageing may have distinct dominant mechanisms. In relatively younger older adults, sarcopenia-associated microenvironmental changes are often dominated by mild chronic inflammation, reduced mitochondrial metabolism and resistance to protein synthesis. By contrast, advanced age or frailty is more commonly associated with senescence-like cellular alterations, enhanced SASP activity, impaired mitochondrial quality control and reduced regenerative capacity of satellite cells (148). Recent reviews from a geroscience perspective have highlighted that sarcopenia is associated with multiple hallmarks of ageing, including cellular senescence, mitochondrial dysfunction, chronic inflammation, dysregulated nutrient sensing and stem cell exhaustion. Therefore, age-related heterogeneity should not be assessed solely by chronological age, but should incorporate biological ageing features such as inflammation, metabolism, frailty and regenerative reserve (149). Studies of human and mouse skeletal muscle show enhanced p16^INK4a^, p21^Cip1^ and SASP-associated features in aged skeletal muscle, suggesting that senescent cell accumulation and abnormalities of the local inflammatory niche may predominate in very old individuals. Sex differences are also important determinants of early microenvironmental heterogeneity in sarcopenia. Studies have shown that female rats exhibit more pronounced muscle atrophy and fibrosis during ageing, together with more marked mitochondrial proteomic remodeling, including increased fatty acid oxidation proteins, reduced complex subunits and greater susceptibility to oxidative modification. These changes may be related to weakened estrogen signaling and reduced levels of Parkin, a key mitophagy protein (150).

Nutritional status and metabolic background further shape individual differences in early sarcopenic microenvironmental changes. Insufficient protein intake, vitamin D deficiency and micronutrient insufficiency can impair muscle protein synthesis, antioxidant defense and satellite cell function at an early stage. Evidence suggests that 1,25(OH)_2_D_3_/VDR signaling helps maintain skeletal muscle energy metabolism and proteostasis; its absence can induce muscle metabolic disorders, abnormal insulin regulation and myofiber atrophy (151). In addition, malnutrition, obesity and metabolic abnormalities are often accompanied by gut microbiota dysbiosis. Dysbiosis can affect muscle metabolism and inflammatory status at an early stage by reducing short-chain fatty acid production, enhancing gut-derived inflammation, altering bile acid and amino acid metabolism and disrupting gut–muscle axis homeostasis, thereby further promoting declines in muscle mass and function (152). Therefore, population stratification should consider body mass index, intramuscular fat infiltration, protein intake, vitamin D status and metabolic inflammatory burden in order to distinguish subgroups dominated by nutritional deficiency, metabolic inflammation or regenerative impairment.

Physical activity level also determines the plasticity of early changes in the ageing microenvironment. Sedentary behavior can aggravate disuse muscle atrophy, mitochondrial dysfunction and inflammatory responses, whereas regular exercise can delay muscle ageing through anti-inflammatory, pro-regenerative and metabolic homeostatic effects (153). Systematic reviews and network meta-analyses show that different exercise modalities have varying effects on muscle strength, physical function and muscle mass in older adults with sarcopenia, suggesting that baseline exercise status and exercise responsiveness are themselves components of population heterogeneity (154). Mechanistically, exercise can maintain physical performance and muscle function during ageing through AMPK and mitochondrial dynamics (155). Exercise can also connect mitochondrial function with myofiber type remodeling through ROS-mediated epigenetic regulation, thereby influencing individual adaptability to exercise interventions (156). Therefore, early intervention strategies for sarcopenia should not follow a single uniform model, but should be stratified according to age, sex, nutritional background, exercise capacity and dominant microenvironmental alterations, with individualized integrated intervention plans developed accordingly.

### 6.2 Disease-type heterogeneity

The early microenvironmental changes and clinical manifestations of sarcopenia show marked disease-type heterogeneity, largely because different etiologies and comorbid conditions exert distinct effects on inflammation, metabolism, regeneration and matrix remodeling. Primary age-related sarcopenia develops mainly in the context of physiological ageing, and its microenvironmental alterations usually accumulate gradually, involving multiple biological mechanisms of ageing such as chronic low-grade inflammation, cellular senescence, mitochondrial dysfunction, dysregulated nutrient sensing and reduced MuSC regenerative reserve (157). With advancing age, immune dysregulation alters the function and polarization states of immune cells such as macrophages, leading to increased pro-inflammatory mediator release and reduced anti-inflammatory reparative capacity. This immunometabolic imbalance can weaken muscle regenerative capacity before overt muscle mass loss occurs (158). In the aged niche, MuSCs may abnormally exit quiescence or shift toward pre-activated or senescence-like states, accompanied by reduced self-renewal and myogenic differentiation capacity, thereby weakening the functional stem-cell reserve required for muscle repair (159). This slow but persistent microenvironmental imbalance can drive a gradual decline in muscle mass and strength.

By contrast, early microenvironmental alterations in chronic disease-associated secondary sarcopenia are often more complex and progress more rapidly. Sarcopenia secondary to chronic conditions such as diabetes, chronic kidney disease and heart failure is frequently accompanied at an early stage by increased oxidative stress, systemic inflammation, metabolic abnormalities and pro-fibrotic remodeling (160). For example, in type 2 diabetes patients, sarcopenic obesity is associated with higher mortality, with risk exceeding that of patients without either condition (161). These diseases can accelerate early microenvironmental ageing through distinct molecular mechanisms: diabetes- and obesity-related insulin resistance can weaken anabolic signaling pathways such as PI3K/Akt/mTOR, whereas chronic systemic inflammation further promotes protein breakdown, mitochondrial damage and muscle functional decline (162). In addition, non-alcoholic fatty liver disease (NAFLD) has a complex bidirectional relationship with sarcopenia and sarcopenic obesity, involving interactions among adipokines, cytokines, hepatokines and myokines, and may establish a vicious cycle of metabolic inflammation (163).

As a distinct subtype, sarcopenic obesity (SO) is not simply the additive consequence of sarcopenia and obesity. Its early microenvironmental features are characterized by mutually reinforcing adipose tissue inflammation, adipokine and lipid mediator imbalance, a tendency toward intramuscular fat infiltration and insulin resistance. Intramuscular fat infiltration and early lipotoxic changes can disrupt skeletal muscle structural integrity and alter the local microenvironment through inflammatory signaling, oxidative stress and mitochondrial dysfunction, thereby impairing myofiber contractile and metabolic function (162). These structural and metabolic alterations can further weaken myofiber performance through lipotoxicity, inflammatory signaling, oxidative stress and mitochondrial dysfunction (146, 164). This distinctive microenvironmental profile places patients with SO at a higher risk of all-cause mortality (165). Sarcopenia associated with cancer, chronic kidney disease and heart failure is more closely aligned with disease-driven muscle wasting, and its early microenvironmental alterations often occur against a stronger background of systemic inflammation and metabolic disturbance. In cancer cachexia, tumor-derived TNF-α, IL-6 and other factors can activate muscle protein degradation pathways while inhibiting protein synthesis, thereby driving rapid muscle wasting (166). Chronic kidney disease commonly promotes muscle atrophy through protein–energy wasting, declining renal function and secondary metabolic disturbances, and may also bias the assessment of renal function in patients with sarcopenia (167). Sarcopenia is highly common in patients with cardiovascular diseases like heart failure, and is linked to adverse outcomes including mortality, falls, and poorer quality of life (168). Management of disease-associated sarcopenia should not focus solely on muscle mass loss, but should also control the underlying disease, assess nutritional, inflammatory and metabolic status, and integrate resistance exercise, protein and energy support, and comorbidity-specific interventions (169). Therefore, recognizing disease-type heterogeneity in sarcopenia is essential for developing precise individualized treatment strategies.

### 6.3 Tissue and cellular heterogeneity

Skeletal muscle ageing is not a homogeneous process, and different muscle groups show distinct early susceptibilities to ageing and disuse. Studies indicate that lower limb muscles can undergo rapid atrophy during the early phase of disuse, and that the rate of atrophy differs among muscle groups, suggesting that reduced mechanical loading is an important source of early tissue-level heterogeneity (170). These differences may be closely related to variations in weight-bearing and movement patterns during daily activities, which expose different muscle groups to distinct mechanical loads, metabolic demands and regenerative pressures, thereby producing muscle-specific early microenvironmental changes (171). At the myofiber type level, ageing also shows heterogeneity. Type II fast-twitch fibers typically exhibit earlier functional vulnerability and subsequently undergo preferential atrophy, leading to earlier declines in explosive power and rapid movement capacity (172). Muscles enriched in fast-twitch fibers are more prone during ageing to early disruption of satellite cell quiescence maintenance, reduced metabolic adaptability and senescence-like transition (173). Recent studies have shown that the Sirt2–Nur77 axis can regulate satellite cell quiescence and senescence-like changes through epigenetic–metabolic coordination, suggesting that the metabolic state of stem cells within different myofiber niches may influence early muscle-group vulnerability (174). In addition, reduced autophagy and mitochondrial quality control in aged muscle weaken proteostasis and energy homeostasis. P53INP2/TRP53INP2 declines in aged mouse and human skeletal muscle, and enhancement of its autophagy-mediated activity can improve muscle atrophy, mitophagy and ROS accumulation, indicating that impaired protein quality control is an important mechanism linking tissue heterogeneity to muscle functional decline (131). Together, these findings indicate that, owing to differences in function, loading and intrinsic molecular features, different muscle groups undergo varying degrees of microenvironmental remodeling and functional decline during ageing.

The skeletal muscle microenvironment is a complex ecosystem comprising myofibers, MuSCs, immune cells, stromal cells (e.g., FAPs), and vascular cells. Single-cell-resolution studies have revealed that these cellular subpopulations undergo distinct transcriptomic and functional changes during ageing, jointly shaping the pathological microenvironment of sarcopenia (175). For satellite cells, ageing can cause abnormal exit from quiescence or transition into pre-activated or senescence-like states, leading to early impairment of regenerative capacity (176). Studies have shown that aged MuSCs can undergo three-dimensional chromatin reorganization, with rewiring of local chromatin contacts associated with altered transcription factor binding and abnormal gene expression (177). Heterogeneity among stromal and immune cells is equally important. In young muscle, FAPs can support repair, but in the contexts of ageing, chronic inflammation or abnormal injury repair, they may shift early toward pro-fibrotic, pro-adipogenic and dysregulated immunomodulatory states (178). Spatial, temporal and single-cell transcriptomic studies show that FAPs and monocytes/macrophages are key participating cell populations during skeletal muscle injury repair in older adults, and that complement C3-related communication between these populations can affect necrotic myofiber clearance and regeneration (179). Intercellular communication may also change early; for example, extracellular vesicles derived from aged bone marrow mesenchymal stem cells can be taken up by satellite cells and impair their myogenic potential (180). These findings suggest that cellular heterogeneity in skeletal muscle ageing means that sarcopenia is not merely the result of myofiber loss, but rather the combined consequence of dysfunction and disordered interactions among multiple cellular components within the entire muscle ecosystem.

Single-cell omics technologies, particularly single-cell RNA sequencing (scRNA-seq), single-nucleus RNA sequencing (snRNA-seq) and spatial transcriptomics, are revealing cellular heterogeneity within the complex microenvironment of sarcopenia at unprecedented resolution, providing tools for understanding early microenvironmental changes and developing precision intervention strategies. Conventional bulk transcriptomic analyses can obscure intercellular differences, whereas single-cell technologies can resolve the unique gene expression profiles of different cell types in muscle tissue and their dynamic changes during ageing. For example, snRNA-seq is especially suitable for studying skeletal muscle, a multinucleated tissue that is difficult to dissociate, because it enables gene expression profiling from isolated nuclei and comparison of transcriptomic differences between homeostatic and atrophic muscle states (181). These technologies have revealed the heterogeneity and early functional state transitions of satellite cells, immune cells, endothelial cells and stromal cells in aged muscle. By integrating multi-omic data, studies have further identified the role of genomic topology as a regulator of molecular function in aged MuSCs. Spatial transcriptomics adds a spatial dimension, allowing the localization of different cell types within muscle tissue and their interactions to be examined. This is important for understanding neuromuscular junction (NMJ) remodeling, vascular rarefaction and the formation of pro-fibrotic regions (182). For example, these technologies have been used to dissect cell-specific dysfunction networks involving motor neurons, Schwann cells, satellite cells and FAPs in neurogenic sarcopenia (183). Such high-resolution data not only help identify key cellular subpopulations and molecular pathways that drive early microenvironmental changes in sarcopenia, but also provide a foundation for discovering new biomarkers and therapeutic targets. By resolving cellular heterogeneity, early interventions can be designed more precisely, such as specifically targeting pro-inflammatory macrophage subsets, clearing senescence-like FAPs or restoring the regenerative capacity of dysfunctional MuSCs, thereby advancing individualized management of sarcopenia.

### 6.4 Early identification and risk stratification based on the ageing microenvironment

Early identification of sarcopenia is important for delaying disease progression and improving patients’ quality of life. Compared with clinical phenotypes based solely on muscle mass, grip strength or gait speed, ageing microenvironment-related indicators may better identify early pathological changes that occur before marked functional decline. Dysregulated immune homeostasis is one of the earliest and most accessible alterations for monitoring. Mild elevations in circulating inflammatory factors, such as interleukin-6 (IL-6), tumor necrosis factor-α (TNF-α) and C-reactive protein (CRP), can reflect chronic low-grade inflammation, but their specificity is limited and they should not be used alone for early diagnosis of sarcopenia (184). Recent reviews show that patients with sarcopenia have increased IL-6, CRP and TNF-α levels, and that multiple inflammatory, metabolic and muscle-related indicators are associated with muscle mass, muscle strength and physical performance. These findings suggest that inflammatory biomarkers are more appropriate for inclusion in multi-indicator risk stratification systems than as standalone diagnostic markers (185). In addition, peripheral blood immune cell subset analysis can be used to assess immunosenescence. Single-cell studies indicate that ageing is accompanied by remodeling of T cell, B cell, natural killer cell and myeloid lineages. Therefore, combining inflammatory factor measurements with peripheral immune cell subset analysis may help identify individuals at high risk of immunosenescence (186).

In addition to immune-inflammatory changes, mitochondrial dysfunction, increased oxidative stress and failure of cellular quality control are important bases for early sarcopenia risk identification. In aged myofibers, reduced mitochondrial number, abnormal morphology and impaired respiratory chain function can lead to insufficient ATP production and increased ROS generation, thereby weakening the energy supply required for muscle contraction and triggering oxidative damage and catabolic signaling (187). Studies in chronic kidney disease (CKD)-associated muscle wasting models show reduced expression of mitochondrial electron transport chain proteins and decreased oxygen consumption in skeletal muscle, together with increased oxidative damage. ROCK1 activation promotes Drp1 recruitment to mitochondria and induces excessive mitochondrial fission, thereby aggravating muscle atrophy (188). In addition, neuromuscular junction (NMJ) degeneration can be indirectly reflected by biomarkers such as circulating C-terminal agrin fragment 22 (CAF22). Systematic reviews and meta-analyses suggest that elevated CAF levels are associated with sarcopenia, reduced grip strength and lower skeletal muscle index, supporting CAF22 as a candidate biomarker of NMJ instability and muscle functional decline (189, 190). At the same time, reduced autophagy and mitophagy indicate impairment of cellular quality-control systems in aged muscle. Experimental kidney disease models show that enhancing tissue mitophagy can reduce mitochondrial oxidative stress and alleviate muscle wasting, suggesting that ROS, oxidative phosphorylation (OXPHOS) and Parkin-related mitophagy may serve as mechanism-based risk stratification indicators (191). In addition, anabolic resistance is an important early cellular feature of sarcopenia. Insulin/IGF-1 resistance, inflammation and hormonal changes can gradually shift the balance between protein synthesis and degradation towards catabolism (192, 193).

Declines in satellite cell number and function can reflect early deterioration of muscle regenerative reserve, but indicators obtained from muscle biopsy, such as Pax7 expression and the proportion of PAX7/MYOD-positive cells, are more suitable for research-based or mechanistic stratification than for routine clinical screening (194). TNF-α-induced human pluripotent stem cell-derived skeletal muscle organoids can model inflammation-associated sarcopenic phenotypes and show reduced proportions of PAX7^+^/MYOD^+^ satellite cells, suggesting that impaired satellite cell activation in an inflammatory microenvironment may contribute to sarcopenia development (106). Beyond histological indicators, circulating muscle-derived miRNAs, such as miR-1, miR-133 and exercise-responsive miRNAs, may indirectly reflect skeletal muscle homeostasis, metabolic adaptation and regenerative status. Studies show that endurance training-responsive miR-19b-3p can improve skeletal muscle glucose metabolism, suggesting that miRNAs are more suitable as markers for dynamic monitoring and intervention response than as standalone diagnostic criteria (195). In imaging, CT scan quantitatively assess muscle area, muscle index and muscle quality. Recent large-scale studies suggest that CT-derived skeletal muscle area or index, after appropriate adjustment for height, can be used to predict mortality risk, supporting its use as a non-invasive structural indicator in risk stratification (196). Based on these changes, early risk stratification for sarcopenia should move from single-marker judgment toward multidimensional integrated assessment. Clinically, reliance on any single biomarker should be avoided. Instead, inflammatory and nutritional-metabolic serological indicators, immune cell subsets, mitochondrial and autophagy-related molecules, candidate biomarkers such as CAF22 or miRNAs, CT/MRI/ultrasound-based assessments of muscle quality, and functional indicators such as grip strength and gait speed should be integrated to establish a comprehensive system for early identification and risk stratification. Such a system would provide a basis for precision interventions, including exercise, nutritional support, anti-inflammatory therapy and improvement of regenerative function (**Table 2**).

**Table 2:** Early identification markers and risk stratification related to the ageing microenvironment in sarcopenia.

Risk dimension	Main early change	Potential markers	Risk implication	References
Immune-inflammatory imbalance	Chronic low-grade inflammation ↑; immune regulation ↓	IL-6 ↑; TNF-α ↑; CRP ↑; Treg function ↓	Enhanced inflammaging; possible inhibition of muscle regeneration	(184)
Cellular senescence and SASP	Senescent cells ↑; SASP factors ↑	p16 ↑; p21 ↑; IL-6 ↑; IL-8 ↑; MMPs ↑	Amplified senescence signaling; inflammation and tissue degeneration	(17)
Mitochondrial dysfunction and oxidative stress	Mitochondrial function ↓; ROS ↑; oxidative damage ↑	OXPHOS ↓; oxygen consumption rate ↓; ROS ↑; Drp1 ↑	Impaired energy metabolism; promotes myofiber atrophy	(197)
Impaired autophagy/mitophagy	Autophagic flux ↓; clearance of damaged organelles ↓	Atg7 ↓; Parkin-related mitophagy ↓	Reduced cellular quality control; damaged mitochondria accumulation	(131)
Anabolic resistance	Anabolic sensitivity ↓; protein degradation ↑	IGF-1/Akt/mTOR ↓; insulin sensitivity ↓	Imbalanced protein synthesis and degradation; catabolic shift	(198)
Satellite cell dysfunction	Satellite cell number ↓; regenerative capacity ↓	Pax7^+^ cells ↓; PAX7/MYOD-positive cells ↓	Reduced regenerative reserve; impaired injury repair	(82)
Fibrosis and fatty infiltration	ECM deposition ↑; intramuscular fat ↑	Collagen deposition ↑; TGF-β ↑; IMAT ↑	Stiffened muscle microenvironment; reduced contractile efficiency	(89, 199)
Reduced microvascular support	Angiogenic capacity ↓; nutrient supply ↓	VEGFA ↓; capillary density ↓	Insufficient oxygen and nutrient supply; worsened muscle dysfunction	(200)
NMJ degeneration	Denervation ↑; NMJ stability ↓	AChR abnormality; MuSK abnormality; EMG abnormality; muscle strength ↓	Impaired neuromuscular transmission; early strength decline	(201)
Structural and functional decline	Muscle mass ↓; muscle strength ↓; physical function ↓	Imaging assessment; grip strength ↓; gait speed ↓; SPPB ↓	Reflects sarcopenia risk or progression	(157)
Contractile dysfunction	Specific force ↓; excitation–contraction coupling impairment	Grip strength, muscle power, electrophysiological	Early weakness potentially preceding detectable muscle mass loss	(202, 203)

## 7 Therapeutic strategies targeting the ageing microenvironment in sarcopenia

### 7.1 Foundational exercise, nutrition and immune-metabolic reprogramming

The therapeutic logic of sarcopenia should move from simply improving end-stage muscle loss toward early regulation of the ageing muscle microenvironment. Chronic low-grade inflammation, referred to as inflammaging, is closely linked to the development of sarcopenia and is a major factor in muscle wasting. During ageing, immunosenescence and inflammaging reinforce each other, leading to sustained elevation of pro-inflammatory mediators such as TNF-α and IL-6, together with dysregulation of anti-inflammatory signals such as IL-10. These changes disrupt local immune microenvironmental homeostasis in skeletal muscle at an early stage and promote subsequent decline in muscle function (204, 205). Inflammatory imbalance can promote protein degradation through the ubiquitin–proteasome and autophagy–lysosome systems by activating pathways like NF-κB and JAK/STAT (53). At the same time, inflammatory imbalance can disrupt the MuSC niche and suppress satellite-cell activation, proliferation and myogenic differentiation, thereby weakening early skeletal muscle regenerative capacity (82).

Resistance exercise and adequate nutritional support should therefore remain the first-line platform for microenvironment-targeted intervention. Resistance exercise is one of the safest and most accessible non-pharmacological interventions for modulating early dysregulated immune homeostasis. Systematic reviews and meta-analyses have shown that exercise training can reduce IL-6, TNF-α and CRP levels in older adults, although the effects of different exercise modalities on inflammatory mediators are not entirely consistent. This suggests that exercise prescriptions should be individualized according to patients’ inflammatory status, functional level and comorbidity profile (206). Exercise may also promote the transition of macrophages from pro-inflammatory states toward anti-inflammatory and pro-repair states, a phenotypic shift that helps attenuate inflammation-mediated muscle injury and enhance tissue repair (207). In addition, studies in exercise immunology suggest that exercise can limit excessive inflammatory responses and promote mitochondrial and metabolic adaptation by regulating communication among muscle Treg cells, myeloid cells and stromal cells (208). Combined exercise and nutritional intervention is a fundamental strategy for enhancing muscle protein synthesis responses in aged muscle. Resistance exercise and protein supplementation have synergistic effects: exercise increases muscle sensitivity to amino acids, whereas protein supplementation provides substrates for muscle protein synthesis (209). This combined intervention may also improve the early metabolic and inflammatory microenvironment at multiple levels by regulating inflammatory mediators, such as reducing CRP and IL-6 levels, improving insulin sensitivity and enhancing mitochondrial function (210).

Nutritional and metabolic strategies should focus on correcting anabolic resistance and mitochondrial vulnerability rather than simply increasing calorie intake. Increased protein intake can support muscle mass and function, although the effects are influenced by baseline protein intake, exercise status and population characteristics (211). Leucine can activate mTORC1 signaling and promote muscle protein synthesis, but leucine is better regarded as part of high-quality protein or whey protein supplementation rather than as a standalone intervention (212, 213). Vitamin D status can influence skeletal muscle mitochondrial biogenesis and oxidative metabolic capacity, suggesting that vitamin D intervention may be more relevant for early energy metabolism and mitochondrial functional support (214, 215). In selected subgroups, adjunctive approaches such as omega-3 polyunsaturated fatty acids, IL-6/IL-6R–JAK/STAT3 pathway modulation, mitochondria-targeted antioxidants, NAD+ precursor supplementation, autophagy-inducing natural compounds or myostatin pathway inhibition may be explored, but these strategies should be interpreted cautiously because their effects are heterogeneous and disease-context dependent (216–218).

### 7.2 Senescence-targeted therapies and restoration of the regenerative niche

Accumulation of senescent cells is one of the central drivers of the onset and progression of sarcopenia. Through secretion of SASP factors, including pro-inflammatory cytokines, chemokines and matrix-remodeling enzymes, these cells create a detrimental muscle microenvironment that impairs muscle stem cell function and promotes tissue fibrosis. Therefore, targeting senescent cells and their associated phenotypes has emerged as a frontier strategy for sarcopenia treatment. Senolytic agents can selectively induce apoptosis in senescent cells. For example, dasatinib plus quercetin has been shown in preclinical ageing models to reduce senescent-cell burden and improve physical function (219). In models of chronic kidney disease-associated muscle wasting, D+Q also reduces the burden of senescent muscle progenitor cells and their SASP-associated inflammatory mediators, indicating that senolytics may be more appropriate for secondary muscle-wasting subgroups characterized by high senescent cell burden rather than universal interventions for all patients with primary sarcopenia (220).

Senomorphic strategies complement senolytic approaches by suppressing the senescence-associated secretory phenotype without directly eliminating senescent cells. Rapamycin is a classical senomorphic candidate that can reduce SASP factor expression by inhibiting mTORC1 signaling and may improve neuromuscular junction function, thereby delaying sarcopenia progression (221). Natural compounds such as ginsenoside Rh4 may attenuate oxidative stress and inflammatory responses, improve mitochondrial homeostasis through SIRT1-related pathways, reduce senescent cell burden and improve myofiber morphology in D-gal-induced skeletal muscle ageing models; however, these findings remain preclinical and should not be described as evidence of an established sarcopenia treatment (222). Compared with broad senescent cell clearance, future efforts should focus on stratification based on markers such as p16INK4a, p21, SASP profiles and uPAR, enabling selective targeting of pathological senescent cells while preserving senescence-like cells that may exert transient beneficial effects during injury repair (223, 224).

Restoration of muscle satellite cell function and niche support should be integrated with senescence-targeted strategies. During ageing, the efficiency with which muscle satellite cells transition from quiescence to activation can decline early, closely associated with dysregulation of Notch, Wnt, p38 MAPK and mechanotransduction pathways (225). Pharmacological modulation of p38 MAPK activity can reactivate aged muscle satellite cells, and TAZ has been identified as a key regulator of exercise-induced MuSC activation through the Pard3–p38 MAPK–TAZ signaling axis, suggesting that mechanical–transcriptional signaling may be a potential target for treating sarcopenia (226–228). At the niche level, ageing-related ECM remodeling, increased collagen deposition, tissue stiffening, TGF-β activation and FAP dysfunction weaken satellite cell activation, self-renewal and regeneration (229–231). Human umbilical cord mesenchymal stem cells and young extracellular vesicles may improve aged skeletal muscle regeneration through paracrine mechanisms, but their source, tissue specificity and long-term safety remain unresolved (232, 233). The major therapeutic approaches discussed above and their corresponding targets within the proposed early-pathogenesis model are summarized below (**Table 3**).

**Table 3:** Therapeutic strategies mapped to key early pathological alterations in sarcopenia.

Early pathological target	Role in the proposed early-pathogenesis model	Representative therapeutic approaches	Intended therapeutic effect	Ref
Inflammation	Chronic inflammation and impaired immune resolution promote catabolism and regenerative dysfunction	Resistance/aerobic exercise; immune-homeostasis modulation	Reduce inflammaging and restore a pro-regenerative immune environment	(206, 208)
Oxidative stress	Excess ROS reinforces inflammation and contributes to mitochondrial and contractile damage	Exercise; antioxidant and mitochondrial-protective strategies	Reduce ROS and interrupt the inflammatory–oxidative feedback loop	(56, 57)
Cellular senescence	Senescent cells and SASP amplify inflammation, fibrosis and regenerative failure	Senolytics; senomorphics	Reduce senescent-cell burden and suppress SASP signaling	(219, 221)
Satellite-cell dysfunction	Impaired MuSC activation and self-renewal reduce regenerative capacity	Exercise/mechanotransduction; p38 MAPK modulation; niche restoration	Restore MuSC activation, self-renewal and regeneration	(226, 227)
Mitochondrial impairment	Reduced mitochondrial quality and oxidative metabolism increase ROS and energy deficits	Exercise; vitamin D; mitochondrial/metabolic support	Improve mitochondrial function, quality control and energetics	(208, 215)
Contractile dysfunction	Oxidative damage and impaired excitation–contraction coupling reduce force generation	Resistance exercise; reduction of oxidative/mitochondrial injury	Preserve muscle quality and force-generating capacity	(234, 235)

### 7.3 Precision stratification and combination treatment

Precision intervention in sarcopenia should begin with identification of dominant microenvironmental abnormalities before overt muscle mass loss, followed by target matching and dynamic monitoring. Combined measurement of circulating biomarkers related to inflammation, nutritional metabolism and lipid metabolism, together with imaging-based assessment of muscle mass and quality using CT, MRI or ultrasound, can help identify subgroups with early alterations dominated by inflammation, metabolic abnormalities, fatty infiltration tendency or regenerative impairment before marked functional decline occurs (236). Metabolomic and lipidomic studies have identified specific metabolite differences in patients with sarcopenia, which can complement traditional assessments of muscle mass and function and help identify subgroups at early metabolic risk (237). A recent study further identified reduced plasma sarcosine levels in individuals with sarcopenia and showed in aged mice that sarcosine improved muscle regeneration by promoting an anti-inflammatory macrophage state (238). These findings provide further support for linking metabolomic stratification with mechanism-based immunometabolic intervention. Single-cell and single-nucleus RNA sequencing can reveal cell-type-specific molecular alterations during ageing in myofibers, satellite cells, immune cells, FAPs and endothelial cells, thereby helping identify early intervention targets with greater cell-type specificity (108). For example, changes in macrophage phenotype in the skeletal muscle of middle-aged mice may be early drivers of extracellular matrix remodeling and pro-fibrotic alterations, providing potential targets for intervening in the early sarcopenic microenvironment through immunometabolic modulation (239).

**Figure 4:**
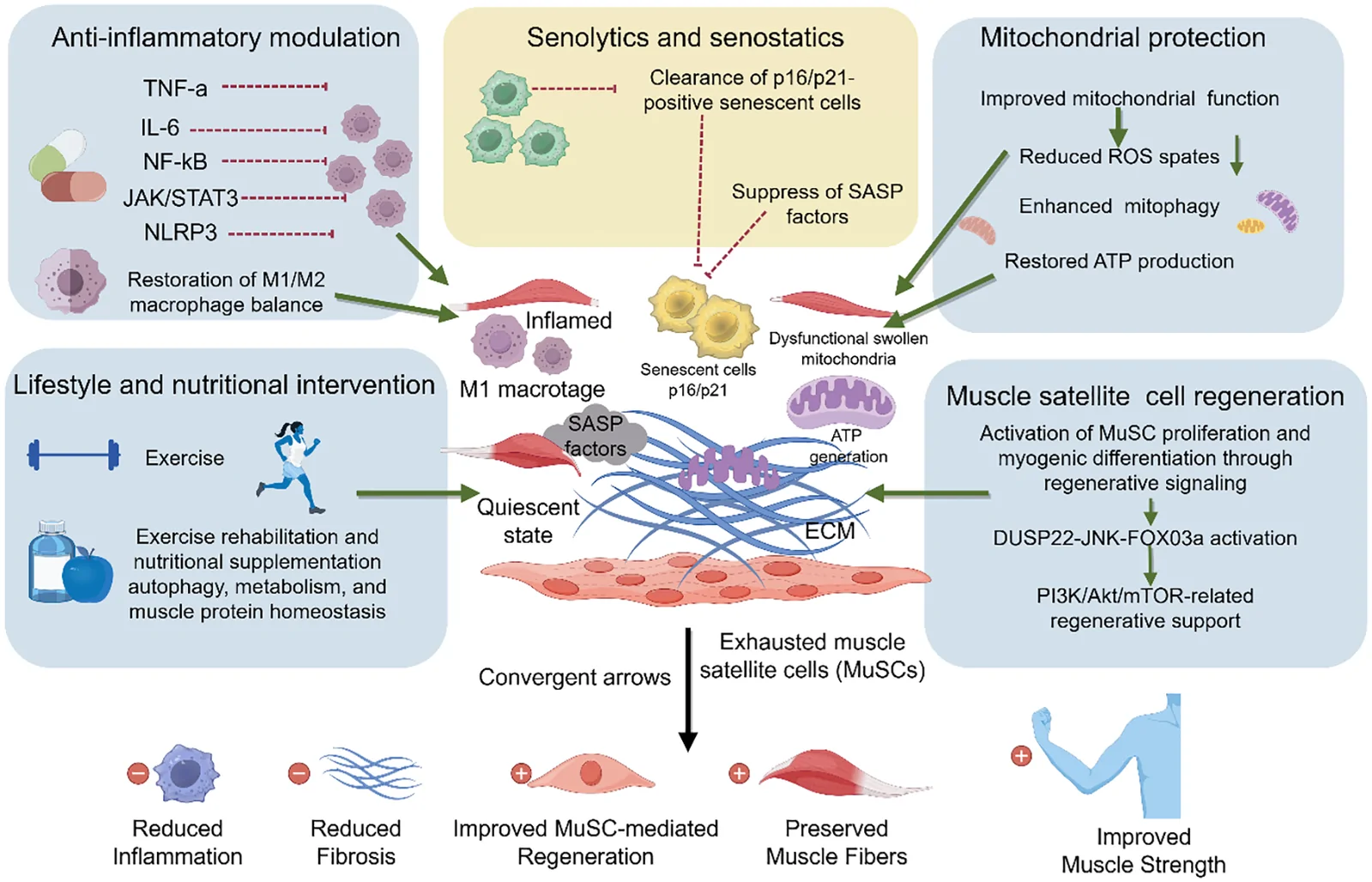
Therapeutic strategies targeting the ageing skeletal muscle microenvironment in sarcopenia. Exercise and nutritional support form the basis of sarcopenia management. Additional strategies include modulation of inflammation and cellular senescence, restoration of mitochondrial function and proteostasis, and improvement of the MuSC niche, vascular support and neuromuscular integrity. Biomarker- and omics-based stratification may facilitate individualized intervention. By Figdraw (www.figdraw.com).

On this basis, intervention should be layered rather than simply additive. Exercise and nutrition should serve as the foundation; anti-inflammatory or immune homeostasis-modulating interventions may be considered for individuals with high inflammatory burden; senolytics or senomorphics may be explored for those with prominent senescent cell or SASP features; metabolic and quality-control interventions should be strengthened in individuals with mitochondrial dysfunction and proteostatic imbalance; and remodeling of the satellite cell niche should be prioritized in those with marked early regenerative impairment. Higher levels of physical activity are associated with lower sarcopenia risk, whereas sedentary behavior and low activity levels are associated with reduced muscle strength and physical performance, suggesting that exercise prescriptions should be dynamically adjusted according to baseline function, fall risk, comorbidity status and tolerance (240). Overall, precision intervention should be based on biomarkers, imaging, omics and functional assessment to achieve stratified identification, target matching, dynamic monitoring and combined intervention before overt muscle mass loss, thereby establishing a continuous closed-loop management model (**Figure 4**).

### 7.4 Challenges to clinical translation

Despite the therapeutic potential of strategies targeting the ageing skeletal muscle microenvironment, several challenges must be addressed before their clinical translation. First, safety is a major concern, particularly for pharmacological senolytics, immune-modulating agents and regenerative therapies (241). Because cellular senescence, inflammatory responses and stromal-cell activation can also exert transient physiological functions in tissue repair, indiscriminate or prolonged suppression of these processes may cause unintended effects (242). Therefore, future interventions should aim to achieve appropriate dose, treatment duration and target selectivity while carefully evaluating long-term safety in older adults with multimorbidity and polypharmacy. Second, tissue and cell-type specificity remains an important limitation. Many candidate pathways involved in the ageing muscle microenvironment also participate in systemic homeostasis, and their systemic modulation may therefore produce off-target effects (243). Strategies that preferentially target skeletal muscle or specific pathological cell populations, such as pro-inflammatory macrophages, senescence-like FAPs or dysfunctional MuSCs, may improve the therapeutic window (244).

Patient stratification will also be essential in light of the biological heterogeneity of sarcopenia (245). As discussed above, patients may differ in the relative contributions of inflammation, cellular senescence, mitochondrial dysfunction, regenerative failure and metabolic abnormalities. Accordingly, biomarker-, imaging- and omics-based approaches should be used to identify mechanistically defined subgroups and match patients to the most appropriate interventions rather than applying uniform treatment strategies. Clinical trial design should reflect this heterogeneity by incorporating standardized diagnostic criteria, biomarker-enriched recruitment, appropriate target-engagement measures and clinically meaningful outcomes. In addition to changes in muscle mass, future trials should include muscle strength, physical performance and patient-centered functional outcomes, together with sufficiently long follow-up to assess durability and delayed adverse effects (246). Factorial or adaptive trial designs may also be useful for evaluating combinations of exercise, nutritional and mechanism-based therapies while identifying patient subgroups most likely to benefit.

## 8 Conclusions

In summary, this narrative Review summarizes the most widely investigated early alterations in the ageing microenvironment of sarcopenia and their underlying mechanisms. We have focused on the roles of dysregulated immune homeostasis, cellular senescence and the SASP, depletion of muscle satellite cells and disruption of their niche, as well as mitochondrial dysfunction, impaired proteostasis and neuromuscular junction degeneration in the onset and progression of sarcopenia. We further summarize potential intervention strategies targeting these pathological processes. Although chronic low-grade inflammation, senescent cell accumulation, impaired regenerative capacity, and metabolic dysregulation are increasingly recognized as important contributors to sarcopenia progression, major challenges remain, including the lack of validated biomarkers for early detection, an incomplete understanding of population- and tissue-level heterogeneity, and limited translational evidence supporting targeted therapies. In the future, the integration of single-cell sequencing, spatial omics, multi-omics approaches and biomarker-based stratification is expected to further clarify key regulatory nodes within the ageing microenvironment of sarcopenia. Future prospective longitudinal cohort studies will be essential to define the temporal sequence of microenvironmental alterations during sarcopenia development. Repeated assessment of circulating and tissue-based biomarkers related to chronic inflammation, cellular senescence, mitochondrial dysfunction, proteostatic impairment and regenerative capacity, together with serial measurements of muscle strength, mass, quality and physical performance, may help determine whether specific molecular or cellular changes precede clinically detectable functional decline. Such studies should include independent biomarker validation, standardized assays and appropriate adjustment for age, comorbidities, physical activity and nutritional status to distinguish sarcopenia-associated signals from nonspecific features of ageing. Importantly, temporal associations observed in longitudinal cohorts would strengthen causal inference but would not by themselves establish causality. Integration of longitudinal human data with mechanistic and interventional studies will therefore be required to determine whether specific microenvironmental alterations directly contribute to disease progression or primarily represent downstream consequences or correlates of sarcopenia.

These advances may also enable rational combinations of exercise and nutritional interventions, inflammatory modulation, senescent cell clearance or SASP inhibition, improvement of mitochondrial function and restoration of the muscle satellite cell niche. Overall, ageing is a natural component of the human life course, and the onset and progression of sarcopenia are closely linked to continuous remodeling of the ageing skeletal muscle microenvironment. Therefore, modulation of the ageing microenvironment may help shift sarcopenia prevention and treatment from simply improving end-stage declines in muscle mass and strength toward early identification, precision intervention and individualized management. From an integrated perspective, this approach provides new therapeutic insights for delaying muscle functional decline and improving healthspan in older adults.

## Glossary

SASP: senescence-associated secretory phenotype
MuSCs: muscle satellite cells
FAPs: fibro/adipogenic progenitors
ECM: extracellular matrix
NMJ: neuromuscular junction
IMAT: intermuscular adipose tissue
SO: sarcopenic obesity
IL: interleukin
TNF-α: tumor necrosis factor-α
IFN-γ: interferon-γ
Treg cells: regulatory T cells
IgG: immunoglobulin G
cTnT: cardiac troponin T
GDF15: growth differentiation factor 15
MANF: mesencephalic astrocyte-derived neurotrophic factor
DAMPs: damage-associated molecular patterns
ATP: adenosine triphosphate
DNA: deoxyribonucleic acid
mtDNA: mitochondrial DNA
HMGB1: high mobility group box 1
TLRs: Toll-like receptors
NLRP3: NOD-like receptor family pyrin domain containing 3
NF-κB: nuclear factor kappa-B
JAK: Janus kinase
STAT3: signal transducer and activator of transcription 3
IL-6R: interleukin-6 receptor
TRAF3: tumor necrosis factor receptor-associated factor 3
MAPK: mitogen-activated protein kinase
p38 MAPK: p38 mitogen-activated protein kinase
FoxO: forkhead box O
IGF-1: insulin-like growth factor 1
PI3K: phosphoinositide 3-kinase
Akt: protein kinase B
mTOR: mechanistic target of rapamycin
mTORC1: mechanistic target of rapamycin complex 1
AMPK: AMP-activated protein kinase
MuRF1: muscle RING-finger protein 1
MAFbx: muscle atrophy F-box
GSDMD: gasdermin D
MMPs: matrix metalloproteinases
CCL2: C-C motif chemokine ligand 2
MCP-1: monocyte chemoattractant protein-1
TGF-β: transforming growth factor-β
C3: complement component 3
C4b: complement component 4b
ROS: reactive oxygen species
OXPHOS: oxidative phosphorylation
CMA: chaperone-mediated autophagy
EVs: extracellular vesicles
miRNAs: microRNAs
mRNA: messenger RNA
scRNA-seq: single-cell RNA sequencing
snRNA-seq: single-nucleus RNA sequencing
SGCA: alpha-sarcoglycan
HIF-1: hypoxia-inducible factor 1
AP-1: activator protein-1
cGAS: cyclic GMP-AMP synthase
STING: stimulator of interferon genes
p16INK4a: cyclin-dependent kinase inhibitor 2A
p21CIP1/CDKN1A: cyclin-dependent kinase inhibitor 1A
SA-β-Gal: senescence-associated β-galactosidase
uPAR: urokinase-type plasminogen activator receptor
CAR-T: chimeric antigen receptor T cells
Pax7: paired box 7
MyoD/MYOD: myogenic differentiation 1
ERK1/2: extracellular signal-regulated kinases 1/2
DRP1/Drp1: dynamin-related protein 1
PDGFRα: platelet-derived growth factor receptor-α
C/EBPδ: CCAAT/enhancer-binding protein δ
LOXL2: lysyl oxidase-like 2
FGF7: fibroblast growth factor 7
FGFR2: fibroblast growth factor receptor 2
Smoc2: SPARC-related modular calcium-binding protein 2
YAP: Yes-associated protein
TAZ: transcriptional coactivator with PDZ-binding motif
VDR: vitamin D receptor
NAD+: nicotinamide adenine dinucleotide
NR: nicotinamide riboside
NMN: nicotinamide mononucleotide
SIRT1: sirtuin 1
SIRT3: sirtuin 3
MitoQ: mitochondria-targeted ubiquinone
OLE: oleuropein aglycone
CCFE: Castanea crenata flower extract
EPA: eicosapentaenoic acid
DHA: docosahexaenoic acid
D+Q: dasatinib plus quercetin
CKD: chronic kidney disease
NAFLD: non-alcoholic fatty liver disease
CRP: C-reactive protein
CAF: C-terminal agrin fragment
CAF22: C-terminal agrin fragment 22
ActRIIB-Fc: activin type IIB receptor-Fc fusion protein
CT: computed tomography
MRI: magnetic resonance imaging
SPPB: Short Physical Performance Battery.
